# Complete genome of *Staphylococcus aureus* Tager 104 provides evidence of its relation to modern systemic hospital-acquired strains

**DOI:** 10.1186/s12864-016-2433-8

**Published:** 2016-03-03

**Authors:** Richard W. Davis, Andrew D. Brannen, Mohammad J. Hossain, Scott Monsma, Paul E. Bock, Matthias Nahrendorf, David Mead, Michael Lodes, Mark R. Liles, Peter Panizzi

**Affiliations:** Department of Drug Discovery and Development, Harrison School of Pharmacy, Auburn University, 4306 Walker Building, Auburn, AL 36849 USA; Department of Biological Sciences, Auburn University, 101 Rouse Life Science Building, Auburn, AL 36849 USA; Lucigen Corporation, 2905 Parmenter St, Middleton, WI 53562 USA; Department of Pathology, Microbiology, and Immunology, Vanderbilt University Medical Center, Nashville, TN 37232 USA; Center for Systems Biology, Massachusetts General Hospital and Harvard Medical School, Simches Research Building, 185 Cambridge St., Boston, MA 02114 USA

## Abstract

**Background:**

*Staphylococcus aureus* (*S. aureus*) infections range in severity due to expression of certain virulence factors encoded on mobile genetic elements (MGE). As such, characterization of these MGE, as well as single nucleotide polymorphisms, is of high clinical and microbiological importance. To understand the evolution of these dangerous pathogens, it is paramount to define reference strains that may predate MGE acquisition. One such candidate is *S. aureus* Tager 104, a previously uncharacterized strain isolated from a patient with impetigo in 1947.

**Results:**

We show here that *S. aureus* Tager 104 can survive in the bloodstream and infect naïve organs. We also demonstrate a procedure to construct and validate the assembly of *S. aureus* genomes, using Tager 104 as a proof-of-concept. In so doing, we bridged confounding gap regions that limited our initial attempts to close this 2.82 Mb genome, through integration of data from Illumina Nextera paired-end, PacBio RS, and Lucigen NxSeq mate-pair libraries. Furthermore, we provide independent confirmation of our segmental arrangement of the Tager 104 genome by the sole use of Lucigen NxSeq libraries filled by paired-end MiSeq reads and alignment with SPAdes software. Genomic analysis of Tager 104 revealed limited MGE, and a νSaβ island configuration that is reminiscent of other hospital acquired *S. aureus* genomes.

**Conclusions:**

Tager 104 represents an early-branching ancestor of certain hospital-acquired strains. Combined with its earlier isolation date and limited content of MGE, Tager 104 can serve as a viable reference for future comparative genome studies.

**Electronic supplementary material:**

The online version of this article (doi:10.1186/s12864-016-2433-8) contains supplementary material, which is available to authorized users.

## Background

The prevalence of methicillin-resistance in *Staphylococcus aureus* (*S. aureus*) is a global threat as noted by the Centers for Disease Control and Prevention 2013 Threat Report [1]. *S. aureus* strains are heterogeneous, and therefore can cause a wide range of localized infections, such as impetigo and cellulitis, to more serious systemic infections, such as bacterial endocarditis and sepsis. In particular, *S. aureus* is the leading cause of bacterial endocarditis, a disease with mortality rates as high as 25–47 %, even in the presence of antibiotic therapy [2]. This wide range of infections, as well as the growing number of pathogens with diverse antibiotic resistance profiles, is due to the acquisition of mobile genetic elements (MGEs) that grant heterogeneity to *S. aureus* strains. The best described of these MGEs is the Staphylococcal Cassette Chromosome *mec* (SCCmec) operon, which encodes the *mecA* gene and confers methicillin resistance [3]. However, many other MGEs can confer adaptive advantages during infection, such as enterotoxins [4], leukocidins (most prominently the Panton-Valentine leukocidin, *pvl*, encoded by the *lukF-PV* and *lukS-PV* genes) [3, 4], staphylokinase (*sak*) [5], and the toxic shock syndrome toxin-1 [6]. Therefore, there is an urgent need to better understand the evolutionary adaptations commonly associated with acquisition of MGE that contribute to (i) the prevalence of nosocomial infections, (ii) the rapid spread of community-acquired strains, (iii) development and expansion of novel resistance mechanisms, and (iv) natural host selection and propagation pathways.

To juxtapose recent next-generation sequencing efforts with pathogenic potential, it is necessary to have several well-characterized reference strains to use as landmarks to interpret MGE findings. This is currently lacking in the field, as the most popular strains (i.e. methicillin-sensitive *S. aureus* (MSSA) Newman [7], methicillin-resistant *S. aureus* (MRSA) USA300 [8], and vancomycin-resistant *S. aureus* (VRSA) Mu50 [4, 9]) are complicated by horizontal gene transfer of MGE and inclusion of various prophages that were gained to most likely provide some sort of survival advantage. As such, it is prudent to define strains with a balance between limited MGE presence and potent pathogenic potential.

Here we describe one such strain, which we term *S. aureus* Tager 104. Tager 104 was originally isolated at the New Haven Hospital (New Haven, Connecticut) by Morris Tager et al. in 1947 from a patient with a cutaneous infection caused by a hemolytic bacteria [10, 11]. As a comparison, the first strain of methicillin-resistant *S. aureus* was isolated in 1961 [3]. Morris Tager and co-workers subsequently demonstrated that *S. aureus* Tager 104 induced clotting through expression of secreted factors [12], developed staphylocoagulase purification protocols [13], and initiated preliminary characterization of staphylocoagulase function in contrast to normal physiologic clotting [14, 15]. Currently, staphylocoagulase from *S. aureus* Tager 104 is arguably the most well characterized prothrombin activator studied to date, as it was the original source used to solve the staphylocoagulase (1–325) fragment crystal structure in complex with both thrombin and its immediate precursor, prethrombin 2 [16]. This recombinant N-terminal fragment of staphylocoagulase from Tager 104 binds with high affinity (*K*_D_ 17–72 pM) to the human prothrombin zymogen [17], which was also used to characterize fibrinogen recognition by the prothrombin-staphylocoagulase complex and to determine that two prothrombin-staphylocoagulase complexes bind to a single substrate fibrinogen molecule to mediate cleavage [18]. With the wealth of biochemical insight that has been generated from Tager 104 virulence factors, the genomic characterization has merit from both clinical and evolutionary perspectives.

## Methods

### Ethics statement

For this study, C57BL/6 mice were purchased from The Jackson Laboratories (Bar Harbor, ME). Mice were housed at Auburn University College of Veterinary Medicine with *ad libitum* access to alfalfa-free chow and water. All procedures were designed in accordance with the Guide for the Care and Use of Laboratory Animals of the National Institutes of Health and approved by the Institutional Animal Care and Use Committee of Auburn University for this sepsis model under protocol 2014–2427. *S. aureus* strain Tager 104 was obtained as a depersonalized human isolate from an outside source and therefore no ethics approval was required for its use in this study.

### Mouse model of systemic infection

For this experiment, 12 female C57BL/6 were anesthetized with 1-3 % isoflurane mixed with medical grade oxygen using a vaporizer. *S. aureus* Tager 104 was grown overnight at 37 °C in 50 mL of brain-heart infusion (BHI) broth in a 125 mL Erlenmeyer flask. Cells were twice washed with 40 mL of filter-sterilized phosphate buffered saline (PBS) supplemented with 10 % glycerol. Cells were pelleted by centrifugation at 10,000 × *g* for 10 min and the cell pellet was re-suspended in a final volume of 10 mL of PBS with 10 % glycerol. A sample from this inoculum stock was diluted 1:100 in PBS with 10 % glycerol in a cuvette for determination of cell density by measuring the absorbance at an optical density (O.D.) at 600 nm using a Beckman Coulter DU800 spectrophotometer. These freshly prepared inoculums were injected into these 12 female C57BL/6 mice by tail-vein at a dose of 5 × 10^8^ colony forming units (CFU). After 24 h, the animals were euthanized.

### Histology

Organs were harvested, stored in 4 % paraformaldehyde, and embedded in optimal cutting temperature (OCT) medium by immersing in 4-methylbutane in a metal canister within a dry-ice bath. Slices (10 microns in width) were made using a Thermo HM550 Cryostat set at −19 °C, and fixed to poly-L-lysine coated slides. Slides were then stained using standard Gram and hematoxylin and eosin (H&E) staining protocols. Slides were viewed at 100 or 400 times total magnification on a Zeiss Axioskop 40 microscope, and images were taken using a Nikon DS-Fi1 camera head and DS-L3 control unit.

### Lucigen NxSeq library construction

Genomic DNAs were prepared by E.Z.N.A. Bacterial D.N.A. kit (Omega Bio-tek, Norcross, GA). All genomic DNA preparations were evaluated for approximate size and integrity by Pulsed Field Gel Electrophoresis with a BioRad CHEF-DR III instrument. Fragment Libraries were constructed with a NxSeq DNA Sample Prep Kit (Lucigen). Briefly, genomic DNAs were sheared to approximately 500 to 700 bp with a Covaris instrument and fragment size was confirmed by agarose gel electrophoresis. Five hundred ng sheared DNA was mixed with 2X buffer and a cocktail of end repair and tailing enzymes and then incubated at 25 °C for 20 min and then 72 °C for 25 min. Illumina sequencing adaptors and ligase were then added and the tubes were incubated at 25 °C for 30 min prior to size selection with Agencourt AMPure XP beads (Beckman Coulter). Fragment libraries were then quantified with a 2100 Bioanalyzer (Agilent Technologies), sequenced on a MiSeq Sequencer (Illumina) and 2 × 250 paired end reads were exported for analysis.

### Mate pair libraries

Insert preparation, size selection and library construction followed the NxSeq® 8 kb Long Mate Pair Library Kit. Briefly, 8 kb mate pair library insert was sheared to approximately 10 kb with either a Megaruptor (Diagenode Inc., Denville, NJ) or a g-TUBE (Covaris, Woburn, MA). Sheared DNA was bead-cleaned and then size-selected with Agencourt AMPure XP beads. After size selection and quantification with a Qubit 2.0 Fluorometer (ThermoFisher), 400 ng of insert was ligated to a coupler at a concentration of 1 ng/μL for 16 h at 16 °C. Unligated DNA was removed by digestion with exonucleases and the circularized DNA was purified with Agencourt AMPure XP beads. Purified DNA was then digested with endonucleases (ThermoFisher) to remove DNA between the two ditags, and the ditag-ligated coupler was purified by biotin capture with MyOne C1 streptavidin magnetic beads (ThermoFisher). The purified ditag-ligated coupler was then G-tailed and ligated to a C-Tailed Junction Code adaptor. After bead cleaning to remove unligated adaptor, the Junction Code adapted DNA was re-circularized at low concentration, cleaned with Agencourt AMPure XP beads and then amplified with Accura HF Hot Start Master Mix (Lucigen) and indexed primers.

Construction of 20 kb mate pair libraries followed the NxSeq® 20 kb Long Mate Pair Library Kit protocol. Briefly, 15 μg of high molecular weight genomic DNA was sheared to 20 kb with a g-TUBE at 4500 rpm, end repaired, A-tailed and ligated to adaptors prior to precipitation and resuspension in 10 mM Tris. Adapted insert was gel-isolated either with a BluePippin instrument (Sage Science) or by separation on a 0.3 % SeaKem Gold agarose (Lonza) followed by electroelution with an EluTrap device (Whatman). Gel-isolated insert was then quantified by Qubit and 1 μg of insert was ligated to a coupler at a concentration of 1.25 ng/μL for 16 h at 4 °C. Unligated DNA was removed by digestion with exonucleases and the circularized DNA was purified by alcohol precipitation. The 20 kb protocol continues with endonuclease digestion as described above for the 8 kb mate pair protocol. Mate pair libraries were quantified with a 2100 Bioanalyzer, sequenced on a MiSeq Sequencer (Illumina) and 2 × 250 paired end reads were exported for subsequent filtering and trimming.

True mate pairs were filtered by analysis and trimming of Chimera Code coupler sequences using the Chimera-Clean5 Python script (Lucigen); junctions between left and right di-tags were identified by presence of the Junction Code linker and di-tags extracted using the JunctionSplit9 Python script (Lucigen).

### Tager 104 genome construction

Genomic DNA was extracted from Tager 104 using E.Z.N.A. Bacterial D.N.A. kit (Omega Bio-tek, Norcross, GA) and constructed into a bar-coded library using the Nextera DNA sample preparation kit (Illumina, San Diego, CA). Sequencing was performed using an Illumina MiSeq sequencer for 2 × 150 paired end reads and trimmed sequence reads were assembled *de novo* using CLC Bio v. 4.6.1, as described previously [19]. To scaffold these contigs, a sub-library of Tager 104 was constructed for PacBio SMRT sequencing. Two sequencing reactions were performed, and CLC bio contigs were scaffolded using Celera Assembler pipeline on the SMRT analysis 1.3 suite [19].

To overcome the innate difficulty in genome closure in this initial construction, PacBio reads were instead assembled *de novo* using SMRT Analysis v. 2.0 Hierarchical Genome Assembly Process (HGAP) algorithm [20], which produced 8 contigs. To close the genome, two Lucigen NxSeq 20 kb mate-pair libraries were constructed and sequenced on an Illumina HiSeq system. PacBio HGAP scaffolds and Lucigen NxSeq paired-end reads from the mate-pair library were provided to SSAKE-based scaffolding of Pre-Assembled Contigs after Extension (SSPACE) [21] for *de novo* assembly to create the closed draft genome. Gap regions in this genome were closed using a combination of the GapFiller algorithm (as part of the SSPACE suite) and Basic Local Alignment Search Tool (BLAST) search against the initial CLC contigs for those which bridge gap regions. This complete, circular genome was submitted to the Rapid Annotation using Subsystem Technology (RAST) server [22–24].

To confirm the construction of Tager 104 using an independent method, new libraries were constructed using the Nextera DNA kit and sequenced using Illumina MiSeq 2 × 250 reactions. These results were combined using the St. Petersburg genome assembler (SPAdes) algorithm [25] for genome closure.

### Construction validation and analysis

To test the contribution of repeats to the shortcomings in genome assembly, long repeats (>500 bp) were identified using Nucmer mapping of the Tager 104 genome to itself [26] and selecting for regions with unique locations and proper size. In addition, interspersed repeats and RNA sequences were identified using the RepeatMasker algorithm (www.repeatmasker.org). The coordinates of unique repeats were recorded and provided to Circos version 0.64 (www.circos.ca).

To determine the contribution of Illumina MiSeq contigs (constructed using CLC), PacBio RS sequencing reads, and scaffolds constructed from the combination of the two, results from each assembly were mapped to the Tager 104 genome using Nucmer. The locations of each unique mapping were provided to Circos for visualization.

### MLST analysis

To compare the lineage of Tager 104 to other clinical *S. aureus* strains, we employed multi-locus sequence typing (MLST). FASTA-formatted genomic sequences of completed *S. aureus* genomes were obtained from the RefSeq database on GenBank and uploaded to CLC Workbench. The identity of the seven housekeeping genes (carbamate kinase, *arc;* shikimate dehydrogenase, *aro*; glycerol kinase, *glp*; guanylate kinase, *gmk*; phosphate acetyltransferase, *pta*; triosephosphate isomerase, *tpi*; acetyl coenzyme A acetyltransferase, *yqi*) were determined *in silico* using the MLST module (http://www.clcbio.com/clc-plugin/mlst/). The sequence type of Tager 104 was determined previously to be ST49, as outlined elsewhere [19].

### Proteomic analysis of Tager 104 genome

To perform whole-genome comparisons of Tager 104 to reference *S. aureus* strains, a CMG-Biotools virtual machine was graciously provided by Dr. David Ussery [27]. One reference genome was selected from each ST group represented in MLST analysis. Each genome sequence was downloaded from GenBank using the getgbk (*i.e.*, content) function, and converted into FASTA files using saco_convert. These files were then submitted to the prodigalrunner algorithm to process the open reading frames and create a protein FASTA file. The makebmdest and blastmatrix algorithms were then utilized to construct BLAST matrices using one representative from each ST.

### Whole-genome phylogenetic analysis

The Tager 104 genome and the GenBank reference sequence genomes were obtained from RAST and GenBank, respectively. Genomes were submitted to alignment by the Mauve algorithm using default parameters [28]. Orthologs were extracted from the resulting .xmfa file using stripSubsetLCBs (http://darlinglab.org/mauve/snapshots/2015/2015-01-09/linux-x64/) with the minimum locally colinear blocks length set to 500 bp and the number of genomes set to 75. The result was then converted to FASTA format using the xmfa2fasta.pl script, and the FASTA-formatted alignment was then converted to phylip format using the fasta2phylip.pl script (both parts of the BioPerl package) (www.bioperl.org). To construct the phylogenetic tree, a maximum-likelihood tree was constructed using the RAxML program on the Cyberinfrastructure for Phylogenetic Research (CIPRES) Science Gateway using default settings (www.phylo.org/portal2/home.action), and the image was constructed using FigTree (tree.bio.ed.ac.uk/software/figtree).

### MGE-encoded gene determinations

The list of MGE-encoded predicted virulence factors was determined based on previous studies by our lab and others, and reviewed elsewhere [3]. The RAST server was first searched for the presence of each protein-encoding gene (PEG). In cases where no annotated PEG was detected, the UniProt server (www.uniprot.org) was used to obtain DNA sequences for BLAST analysis. The genetic location of these elements was recorded and provided to Circos for graphic representation.

To compare genomes of the Tager clade, we utilized the BLAST Ring Image Generator (BRIG) algorithm (http://brig.sourceforge.net/) with default parameters [29].

Genomic islands were determined using the IslandViewer 3 algorithm, available online at http://www.pathogenomics.sfu.ca/islandviewer. Islands were identified based on their genetic content, as reported by the IslandViewer output and BLAST analysis.

For the νSaβ genomic island, genomic profiles were constructed using the RAST server. After determination of the profile, open reading frames were recolored based on their family or function.

### Antibiotic susceptibility testing

*S. aureus* Tager 104 was streaked for a lawn on trypticase soy agar (TSA) plates containing 5 % sheep blood (BD Biosciences). E-test strips for erythromycin (ER), linezolid (LZ), oxacillin (OX), and vancomycin (VA) (bioMérieux, Inc.) were then placed onto the surface of the agar using sterile forceps. All resistance tests were performed under conditions that were naïve for the *S. aureus* Tager 104 strain to not complicate interpretation by inducing vancomycin-resistance. Bacteria were incubated at 37 °C for 17 h and plates were imaged using an IVIS Lumina XR system. In addition, overnight cultures of *S. aureus* Tager 104 were added at a 1:200 ratio to wells containing BHI broth and serial diluted concentrations of vancomycin. The 96-well plate was incubated for 17 h in an incubator and the O.D._600nm_ was determined using a Thermo Scientific VarioSkan plate reader. Data was subsequently plotted using Graph Pad Prism v4.03.

## Results

### *S. aureus* Tager 104 abscess formation in naïve tissue

**Fig. 1:**
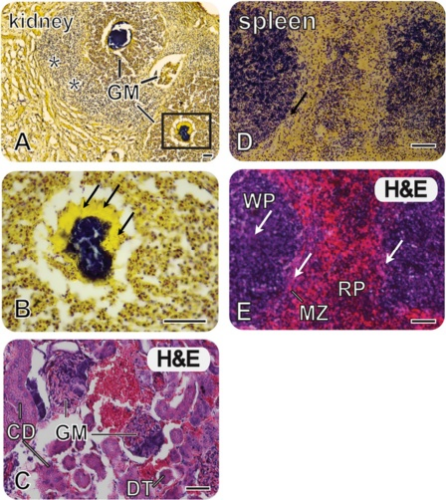
*S. aureus* Tager 104 Bacteremia Leading to Multi-organ Septic Foci. **a** Gram staining of C57BL/6 kidney 8 days after injection of 5 × 10^8^ CFU *S. aureus* Tager 104. **b** Higher magnification (400× total) of the indicated by the box in panel **a**. Arrows indicate the protective fibrin layer that allows the bacteria to thrive in the glomerulus (*GM*) without the threat of clearance by phagocytic immune cells (see area immediately surrounding infected GM shown in Panel **a**). **c** Adjacent section of the same kidney stained with Hematoxylin and Eosin (*H&E*); please note the GM is at a slightly earlier stage of the disease. Kidney architecture including the collecting ducts (*CD*), the distal tubule (*DT*), and blood cells (*red*). **d** Gram staining shows bacteria (*purple*) in all organs examined; splenic abscesses also show thickening of the marginal zone and increased fibrin deposition (arrows). **e** Adjacent section stained for H&E showing the splenic architecture including the white pulp (*WP*) region, marginal zone (*MZ*) and red pulp (*RP*) region

Histology indicates that *S. aureus* Tager 104 is able to colonize all tissue with variable coverage at this time point as indicated by Gram staining (Fig. 1). Bacteria were detected in the alveoli cells of the lung (not shown), the white pulp of the spleen, and glomerulus of the kidney. This supports the previous hypothesis that Tager 104 is capable of systemic spread. Given our previous studies [30, 31], we now know that Tager 104 can cause both endocarditis and establish abscesses in naïve tissue. We sought to better characterize this Tager 104 at a genomic level to consider all potential virulence determinants prior to comparative virulence studies with other *S. aureus* strains.

### Construction of *S. aureus* Tager 104 genome

To close the *S. aureus* Tager 104 genome, three different sequencing technologies were used: Illumina Miseq 2 × 150 bp reactions, PacBio RS 90-min movies, and Lucigen NxSeq 20 kb mate-pair libraries. In all approaches, scaffolds were constructed using one of either Illumina MiSeq or PacBio RS, or a combination of the two (hybrid assembly).

We first sought to close the hybrid assembly scaffolds previously produced for Tager 104 [19]. Therefore, these scaffolds were submitted to the SSPACE algorithm with the Lucigen NxSeq mate pair sequences. This approach successfully closed the genome, producing the 2.8 Mb *S. aureus* genome. However, this approach left six large gaps in the assembly, which would have required significant manual closure.

**Fig. 2:**
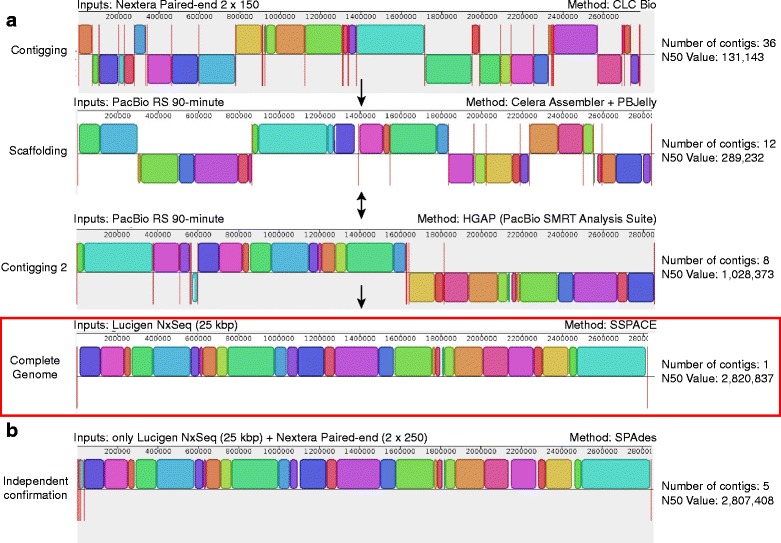
Construction of the Tager 104 Genome. **a** The Tager 104 genome was constructed using indicated methods, with each step incorporating new genomic data (*Inputs*). First, Tager 104 paired-end reads from Illumina MiSeq sequencing were submitted to CLC Bio (*Contigging*). Resulting contigs were scaffolded using reads from PacBio RS sequencing (*Scaffolding*). Due to the inability to further close scaffolds, PacBio RS data alone was instead contigged using HGAP assembly (*Contigging 2*). The eight remaining contigs were then closed using Lucigen NxSeq mate-pair libraries with the SSPACE algorithm. Single-headed arrows represent events where resulting data from the previous step was submitted to algorithms in the subsequent step (*Method*). Steps in which results were interchangeable are indicated by two-headed arrows. The assembly is described in more detail in “Methods.” **b** In order to confirm the completed genome, Lucigen NxSeq libraries were submitted with separate 2 × 250 paired-end reads in an independent SPAdes construction devoid of previous read data from panel *A*. Results were identical to the finished genome

Therefore, we instead tested the ability of PacBio RS sequencing reactions in combination with NxSeq mate-pair libraries. Scaffolds were produced using the Hierarchical Genome Assembly Process (HGAP) assembly algorithm on the PacBio SMRT Analysis suite (v 2.0) [20]. This algorithm produced 8 scaffolds from raw sequencing data, with an N50 value of 1,028,373 bp. These scaffolds were then closed using de-duplicated NxSeq reads using SSPACE. When possible, small gaps were filled by aligning CLC-constructed Illumina contigs to the genome using a BLAST search. The result was a closed 2.82 Mbp genome (Fig. 2a).

To independently confirm our scaffolding of the putative 2.82 Mbp genome, Illumina MiSeq 2 × 250 libraries were constructed and sequenced using Illumina 2 × 250 sequencing reactions, and the resulting contigs were scaffolded using Lucigen NxSeq reactions. The resulting closed genome was identical to that produced by the hybrid HGAP-SSPACE assembly pipeline (Fig. 2b).

### Repeat regions created difficulty in automated closure

**Fig. 3:**
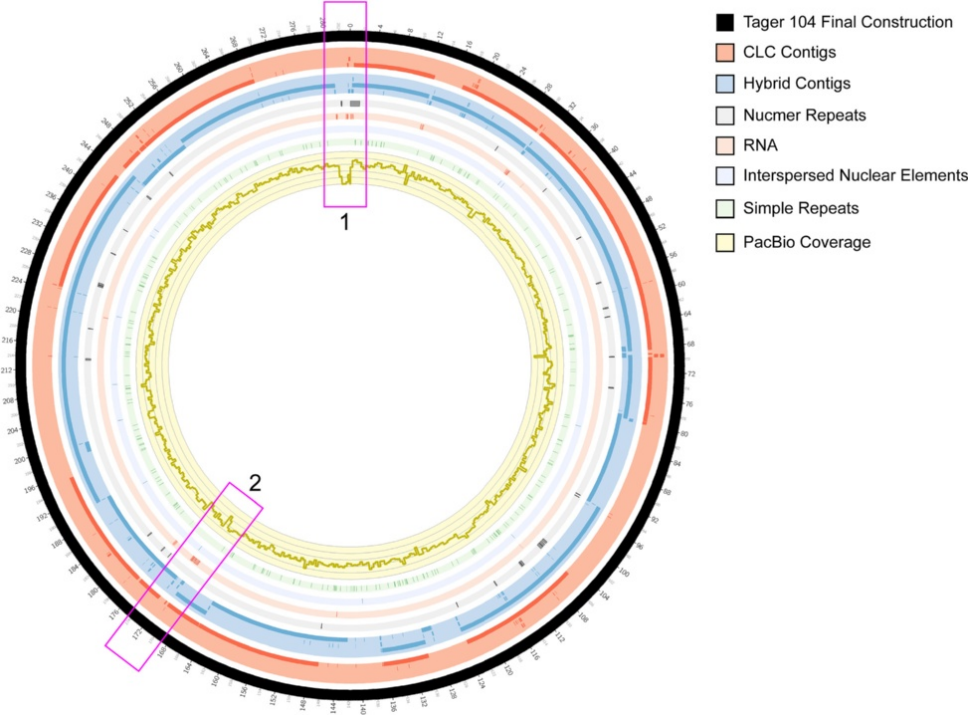
Graphical Depiction of Tager 104 Assembly. The Tager 104 genome was scaffolded using three separate technologies: contigs from MiSeq reactions (*red tiling*), PacBio RS reads (*yellow histogram*), and scaffolds produced from the hybrid assembly of both (*blue tiling*). However, assembly of the Tager 104 genome using these technologies were found insufficient alone, due to the presence of repeat regions. More specifically, long repeats (>500 bp) create errors in de Bruijn graph untangling (*gray tiling*). In addition, the exact placement of short repeats, such as interspersed nuclear elements (*light blue tiling*), RNA sequences (*light red tiling*) and simple repeats (*light green tiling*) create errors in construction. Areas rich in repeats and low in sequencing coverage (*Regions 1 and 2*) reveal difficulties in genomic construction. These difficulties were overcome by providing the construction algorithms with 20 kbp mate-pair information, provided by Lucigen NxSeq libraries

To determine the shortcomings of individual methods on the closing of the Tager 104 genome, as a model for future *S. aureus* genome assembly, each contig or scaffold was mapped to the final genome using Nucmer and visualized using Circos (Fig. 3). In addition, repeats were determined by mapping the Tager genome to itself and selecting for matches with unique locations and a size > 500 bp. Interspersed repeats and RNA sequences were determined using the RepeatMasker algorithm. Based on the analysis, Illumina MiSeq 2 × 150 reads did not properly bridge areas of the genome that were rich in repeats. More specifically, repeats > 500 bp (Fig. 3, Region 1) and RNA repeats (Fig. 3, Region 2) caused the greatest issues in construction, as visualized. In addition, PacBio RS coverage was low across these areas. Therefore, information provided by the Lucigen NxSeq sequencing reactions was essential for the proper untangling and assignment of these genomic regions.

### Proteomic analysis of *S. aureus* reference sequences

Delineation of the *S. aureus* strains is typically achieved using MLST analysis. Briefly, the analysis utilizes sequence heterogeneity among seven housekeeping genes to provide a higher resolution phylogenetic analysis. Reference genomes were obtained from the GenBank RefSeq database (Additional file 1: Table S1). MLST and *spa* typing of *S. aureus* Tager 104 previously revealed it to be ST49, the predicted founder of clonal complex 49 (CC49) [19]. CC49 was also shown to contain ST138, ST1693, ST1937, and ST2273 based on eBurst analysis (data not shown) [32].

**Fig. 4:**
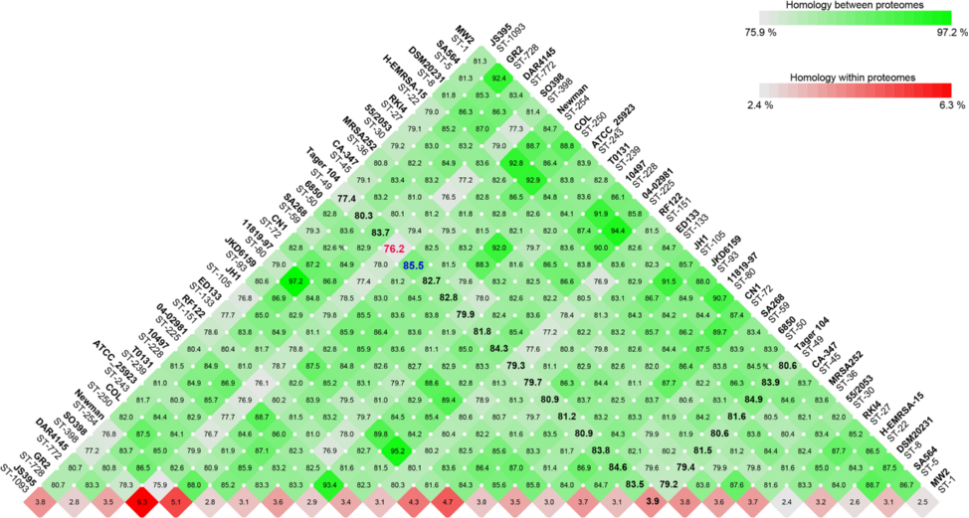
Proteomic Analysis of *S. aureus* Genomes. One *S. aureus* reference sequence GenBank files from each ST group of the completed genomes as of Oct. 2015 were downloaded from the NCBI server and genes were called using the prodigalrunner algorithm on a CMG-Biotools system. The names of the strains are listed with corresponding ST numbers below. Most relevant here, the comparisons to our Tager 104 genome are shown in bold with the highest protein similarity (*blue*) and lowest similarity also shown. Percent paralogs within a given genome are shown at the base of the blast matrix and correspond to the red heat map

To determine the similarities of protein content in completed sequences of *S. aureus*, each genome was called for open reading frames using the prodigalrunner algorithm and compared using the blastmatrix algorithm on CMG-Biotools version 2.2. The results shown in Fig. 4 indicate that the Tager 104 proteome is most closely related to Newman, a strain isolated in 1952 from a human patient and which has been well characterized for its coagulase activity. Similarity in these genomes supports the previously observed similarity in endocarditis models [30]. Following this strain, Tager 104 was > 84 % similar to the nosocomial MRSA strain 04–02981, community-acquired MRSA strain SA268, and the human pleural isolate DSM 20231. The greatest proteomic differences were seen for strain ST398, a typically ovine-associated strain isolated from a human case of endocarditis, which was also the second-largest proteomic divergence observed [33].

### Tager 104 is an ancestor to clinical isolates

To determine the lineage of *S. aureus* reference strains in relation to Tager 104, genomes were submitted to Mauve alignment and conserved segments were stripped and concatenated for each genome for phylogenetic analysis by RAxML. This alignment and phylogeny used only core genomic regions present in all 75 *S. aureus* strains available as of Oct. 1^st^ 2015.

**Fig. 5:**
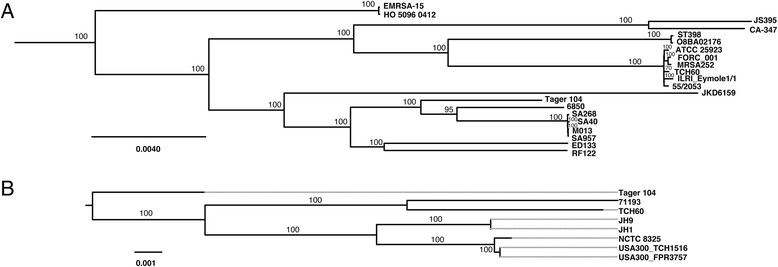
Whole-Genome Phylogenetic Analysis of *S. aureus* Reference Strains. Proximal branches nearest Tager 104 are shown for the phylogenetic analysis of all *S. aureus* reference strains whose genome were available by Oct. 2015 (**a**) or isolates from the United States (**b**) based on Mauve alignment and RAxML analysis. For a complete tree of all strains, please refer to Additional file 2: Figure S1. Bootstrap values represent the result of 100 trials

The resulting tree (Fig. 5 and Additional file 2: Figure S1) shows Tager 104 to be the earliest branching member of a clade containing strain 6850, M013, SA40, SA268, SA957, ED133, and RF122. Strain 6850 was isolated from a skin abscess at the University of Wisconsin in 1987, and was shown to progress from the abscess to complications such as systemic abscesses and septic arthritis [34]. This supports Tager 104 as a reference sequence for systemically-penetrating bacteria. As a positive control, 2 separate genome dataset corresponding to COL and NRS100 were also used and there is indication that these strains are the same or closely related. The results of our analysis are consistent with this hypothesis.

### Tager 104 contains a limited set of MGE-encoded virulence factors targeted at host immunity

**Fig. 6:**
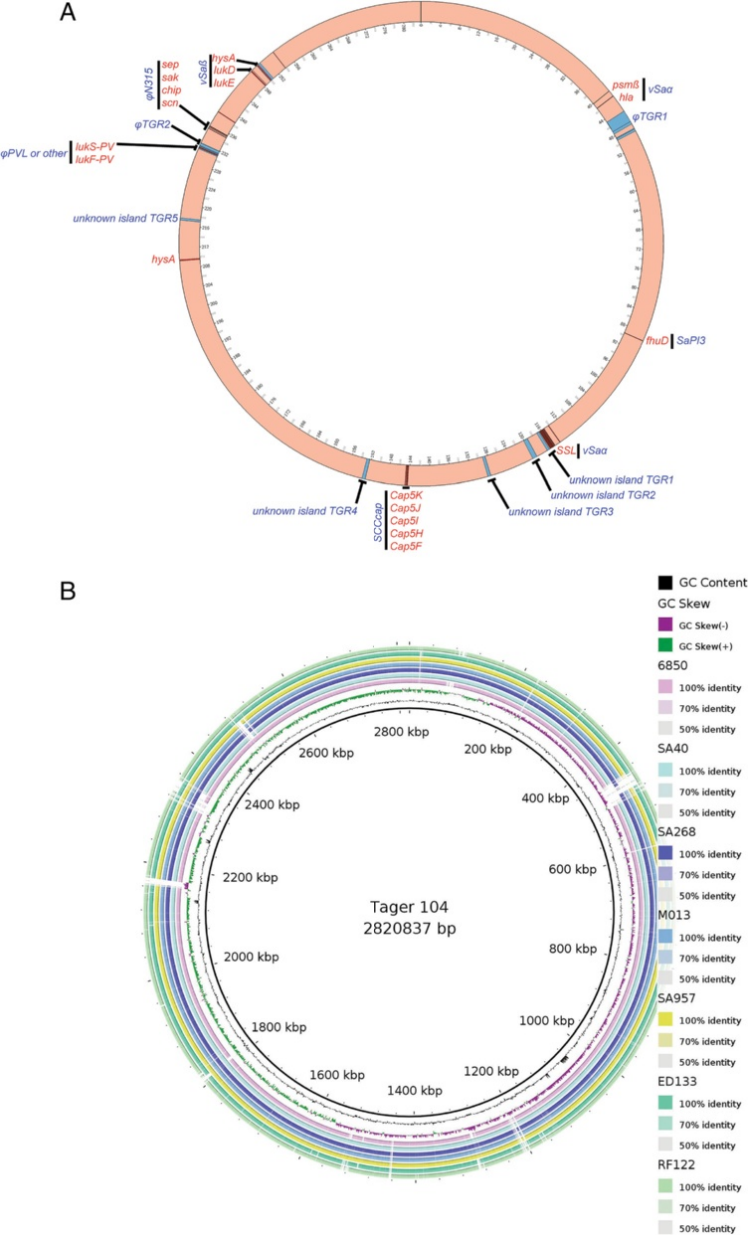
Mobile Genetic Element-Encoded Virulence in *S. aureus* Tager 104. **a** Mobile genetic element (MGE)-encoded genes relevant for virulence in *S. aureus* were selected based on prior knowledge of *S. aureus* genomics. Those elements detected by BLAST analysis in the Tager 104 genome are indicated by locus (*red bands*) as well as gene name (*red labels*), and the genomic island responsible for carrying these elements are grouped (*black bar*) and indicated beside the gene name (*blue label*). In addition, islands were detected using the IslandViewer 3 software, and are indicated by locus (*blue bands*) and given identifiers (*blue label*) based on gene identities and BLAST result. **b** Blast ring analysis of loci from strains in the Tager 104 clade. Tager 104 GC content and GC skew are represented on the innermost ring, with percent identity reported for genomes in the Tager 104 clade reported on the outermost rings as indicated

As Tager 104 was able to survive in septic murine models, and due to its phylogenetic placement as an ancestral *S. aureus* lineage, we investigated the genomic content of MGE-encoded virulence factors that have been previously identified and characterized [3]. The results demonstrate a limited set of encoded factors in the Tager 104 genome that are predicted to be necessary for bacterial survival during bacteremia (Fig. 6a). In addition, BRIG analysis revealed similar genomic regions between strains of the Tager clade (Fig. 6b).

Tager 104 is predicted to encode a νSaα-encoded Staphylococcal super-antigen-like SSL, as well as νSaβ-encoded leukotoxin D and E (*lukD* and *lukE*, respectively). These factors are known to inhibit elements of innate immunity [35]. The νSaβ-island was also shown to contain the hyaluronate lyase gene (*hysA*), which degrades hyaluronic acid found in host extracellular matrix.

The Tager 104 genome also indicates early exposure to phage-carried virulence factors. Tager 104 contained the Staphylococcal inhibitor of complement (*scn*), which targets innate immunity by inhibiting phagocytosis by neutrophils. Tager 104 was shown to contain the *hlb*-interrupting phage described in the genomes of modern clinical strains, such as N315, Mu50, MW2, NCTC8325, MSSA476, MRSA252, USA300, JH1, JH9, and Newman [7], and was defined here as φTGR1. Interestingly, Tager 104 also contains the PVL cassette (*lukF-PV* and *lukS-PV*), a phage-transferred pore-forming leukocyte toxin linked to necrotic infections. Tager 104 only contains one enterotoxin, determined by BLAST analysis as an exact match to the phage-transferred enterotoxin P [3]. PVL-positive *S. aureus* strains are certainly in the minority of the totality of *S. aureus* genomes sequenced to date, but some notable strains include ST1 strains (MW2 and USA400), ST8 strains (NCTC 8325, TW20, USA300, and USA500), and ST239 (TW20).

In addition, Tager 104 was determined to contain one unknown prophage and four unknown genomic islands, indicated as φTGR2 and TGR1 through TGR4, respectively. The contents of these islands were limited to hypothetical and/or phage proteins (Additional file 3: Table S2).

### Tager 104 shows intermediate clinical adaptations

**Fig. 7:**
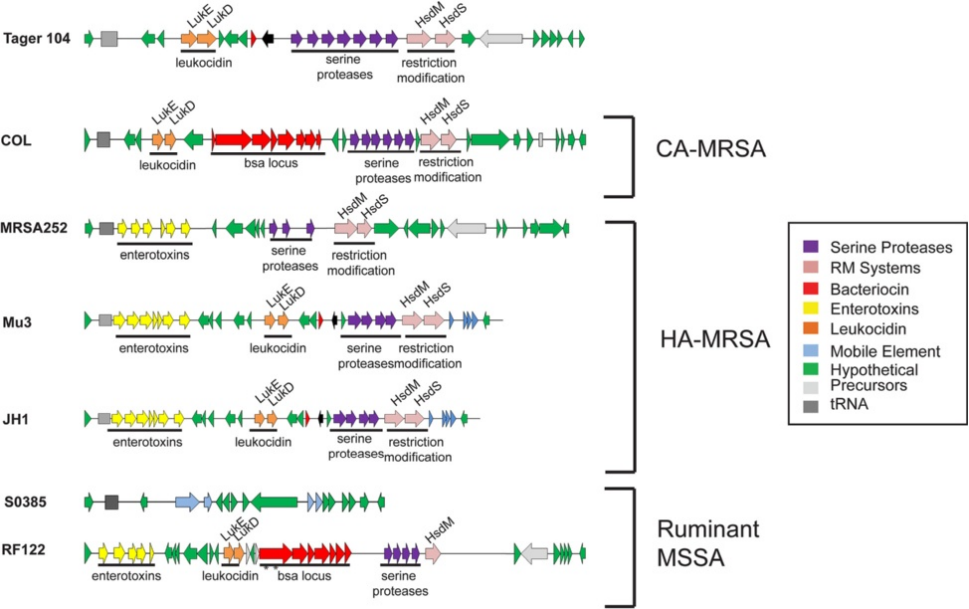
Tager 104 νSaβ Genomic Island Shows Intermediate Clinical Adaptation. Representatives from each subtype of the νSaβ locus were selected and compared using the RAST server. Current strains of community acquired MRSA (*CA-MRSA*) contain the *bsa* locus (*red arrows*), which encodes a bacterial antibiotic. In contrast, hospital-acquired MRSA (*HA-MRSA*) have lost the *bsa* locus and instead gained enterotoxins (*yellow arrows*), an adaptation which has been previously associated with a selection for hospital settings. Results indicate Tager 104 has lost the *bsa* locus, but lacks the enterotoxin cluster (yellow) that has also been associated with this shift. In contrast, ruminant-host MSSA shows a wide range of these genotypes

One group of pathogenicity islands that are non-phage and non-SCC genomic islands are termed νSa islands, and typically contain a combination of virulence factors and either an intact or remnant recombinase [7]. Two such islands, termed νSAα and νSAβ, were previously found to be allelic, and are therefore typed and used in conjunction with sequence type analysis for determination of strain radiation. Comparison between the profiles of the νSAβ genomic island for one community-acquired MRSA (CA-MRSA) strain (COL), three hospital-acquired MRSA (HA-MRSA) strains (MRSA252, Mu3, JH1), and two ruminant host MSSA strains (S0385, RF122), as well as Tager 104 demonstrated that *S. aureus* Tager 104 has lost the majority of the *bsa* locus, with the exception of *bsaG* (Fig. 7). However, *S. aureus* Tager 104 has not acquired enterotoxin genes seen in other strains adapted for the hospital setting (HA-MRSA). The loss of this *bsa* locus, coupled with the gain of enterotoxins, has been previously hypothesized to indicate a shift from the environmental niche to one of hospital acquisition [36]. Therefore, *S. aureus* Tager 104 may represent an intermediate strain for the selection of this νSAβ profile in the hospital setting.

### *S. aureus* Tager 104 antibiotic resistance profile

To determine the potential of Tager 104 for the future study of acquired antibiotic resistance, known MGE-transferred resistance cassettes [3] were searched against the completed genome using BLAST. No resistance elements were detected (data not shown).

**Fig. 8:**
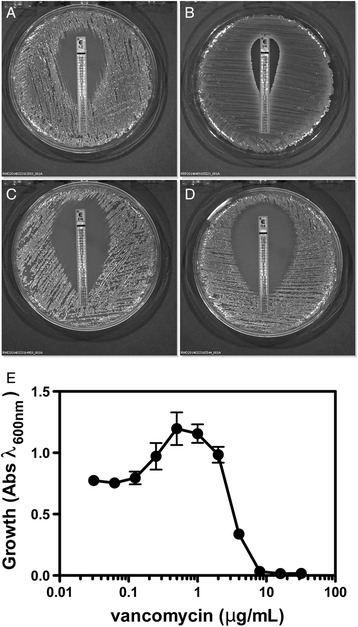
*S. aureus* Tager 104 Susceptibility to Antibiotic Therapy Demonstrates Predation of Resistance Development. **a**–**d** Antibiotic susceptibility testing on sheep blood agar (SBA) plates demonstrated *S. aureus* Tager 104 is susceptible to linezolid (**a**), vancomycin (**b**), erythromycin (**c**), and oxacillin (**d**) when challenged with E-test strips. Minimum Inhibitory concentrations shown are: linezolid, 1 μg/mL; vancomycin, 2–3 μg/mL; erythromycin, 0.064 μg/mL; and oxacillin, 0.019 μg/mL. **e** The effects of vancomycin concentrations on Tager 104 growth were confirmed in solution in 96-well plates and indicate a vancomycin intermediate susceptibility phenotype. This experiment is a combination of 8 replicates and was performed as described in "Methods"

Minimum inhibitory concentrations (MIC) for *S. aureus* Tager 104 were determined by challenging with E-test strips for linezolid, vancomycin, erythromycin, and oxacillin. MICs for antibiotics were determined by E-test strip analysis and *S. aureus* Tager 104 demonstrated resistance to LZ at 1 μg/mL, VA at 2–3 μg/mL, ER at 0.064 μg/mL, and OX at 0.019 μg/mL (Fig. 8). The vancomycin-intermediate susceptibility phenotype of *S. aureus* Tager 104 was confirmed by a solution-based test in a 96-well format; results confirm the MIC to be about 3 μg/mL. *S. aureus* Tager 104 colonies on brain-heart agar plates are well-formed with the absence of satellite projections that would denote the presence of small colony variants and an overall white color, which is unlike other vancomycin-intermediate strains described previously [37].

## Discussion

*S. aureus* infections range in presentation from localized skin infections to more life-threatening osteomyelitis, endocarditis, and sepsis. With the advent of next-generation sequencing, analysis is now possible for individual genomes across the spectrum of infections. These data revealed that certain *S. aureus* strains exhibit considerable changes in their gene make-up, thus reflecting selective pressure to infect a certain host preferentially [38]. The acquisition of MGE and smaller changes in genes known as nucleotide polymorphisms together confer the apparent selective advantages needed for these strains to survive in a given host. The capacity for lateral gene transfer in *S. aureus* strains fuels this potential for rapid adaptation. For example, *S. aureus* Newman, isolated in 1952, has been shown to contain four prophages (φNM1–φNM4) and one additional νSa island, νSa4 [7]. The lack of these prophages was associated with decreased abscess formation in organs. However, a strain such as MW2 lack these prophages, but was isolated from a patient with abscesses of the brain, heart, liver, and kidneys, leading to death [4, 39]. Therefore, the required machinery for a particular infection is highly model-specific, and we suggest that reference strains should be limited in scope to those which suffice for a particular infection model.

Our previous results [30, 31, 40] suggested that *S. aureus* Tager 104 is a potent strain for the formation of bacterial endocarditis, a serious complication of bacteremia. This may not be surprising considering Tager 104 was originally isolated and maintained for decades, due in no small part for its potent ability to form stable clots in human, rabbit, and sheep blood [41]. This clotting phenotype has been attributed to the coordinated effort of two non-proteolytic activators of prothrombin, namely staphylocoagulase [16] and von Willebrand factor binding protein [42, 43]. Both of these factors activate prothrombin by forming a reversible complex that cleaves their substrate fibrinogen to provide the fibrin barrier, similar to those seen here in the focal abscesses in multiple organs, including the kidney and spleen, that were formed by simple induction of the infection by diffuse bacteremia (Fig. 1). Because of these observations, and the early date of its isolation, we sought to outline Tager 104 as a reference strain for the systemic infection niche. We therefore determined the phylogenetic relationship of Tager 104 for future evolutionary and correlation studies compared to other *S. aureus* strains.

Our goal in genome sequencing was to find the simplest and most inexpensive combination of technologies to complete the Tager 104 genome so that it may be used as a reference for future *S. aureus* genomic comparisons. We began with Illumina MiSeq 2 × 150 paired-end libraries, constructed using a Nextera kit. To close the genome, literature suggested that the use of PacBio RS technology would enable bridging across complex regions using longer reads [44]. PacBio reads were corrected using Illumina MiSeq paired-end reads, and scaffolded to create the final genomic construction. The result from this “hybrid assembly” was twelve scaffolds, which have been previously described [19]. However, no combination of algorithms or manual assembly were able to close the genome. New advancements in PacBio construction, ushered by the release of the newer HGAP assembly algorithm, led us instead to reconstruct the genome using raw PacBio data alone, producing 8 scaffolds of varied sizes, which similarly did not result in a complete genome and bridging these contigs manually proved problematic. We therefore decided to approach the closing of the genome using greater distances between the paired reads to provide additional information to the construction algorithms. Two Lucigen 20 kb mate-pair libraries were sufficient to close the Tager 104 genome from this HGAP-assembled data. Furthermore, Illumina 2 × 250 paired-end libraries were constructed, sequenced, and assembled *de novo*. Upon addition of the Lucigen mate-pair libraries, the final genome was produced. Therefore, the aforementioned Lucigen mate-pair library could be used to independently assemble the Tager 104 MiSeq 2 × 250 bp reads. This provides a proof of principle to guide a streamlined protocol for assembly.

To interpret this *S. aureus* Tager 104 genomic dataset in the auspices of the systemic niche, it will need to have the following characteristics: (1) be a clinical sample, (2) be an early branching member of the *S. aureus* phylogenetic tree, and (3) be limited in MGE content to those which are necessary for systemic survival (*i.e.*, immune system evasion). Based on the results of these analyses, Tager 104 has been found to have all of these characteristics. Tager 104 has been maintained from primary freezer stocks since its isolation in the late 1940’s. Tager 104 shows ancestral phylogeny similar to other methicillin-resistant and methicillin-susceptible strains that have been characterized in the literature [45]. This clade is made up of two systemic isolates (6850 and SA957), one wound isolate (M013), one nasopharygenal isolate (SA40), and two ruminant isolates (ED133 and RF122). Interestingly, SA957 occurs later in the lineage than SA40 and has been associated with more severe septic infections and higher mortality rates [46]. This differentiation was hypothesized to be due to the loss of the β-hemolysin-interrupting phage.

Tager 104 shows a limited set of MGE content primarily focused on the evasion of host innate immunity. Tager 104 was shown to contain the *hlb*-interrupting phage described above, labeled as φTGR1, as well as two additional phages. One of these phages contained PVL, indicating the earliest acquisition of this factor in the literature. The other phage was shown to carry no additional genes aside from those necessary for phage construction and replication (Additional file 3: Table S2). For comparison, prophage φTGR1 and φTGR2 were compared proteomically using BLAST matrix analysis with complete phage genomes downloaded from the GenBank server, and showed low homology to three phage genomes (Additional file 4: Figure S2). In addition, Tager 104 was shown to contain the νSaα and νSaβ islands, found in all *S. aureus* genomes [7]. Although Tager 104 was also detected to contain four additional potential genomic islands, their gene content consisted solely of hypothetical proteins. Therefore, the virulence-associated genomic island composition of Tager 104 is limited to those which are found in all genomes (νSaα, νSaβ), the *hlb*-interrupting phage found in most all human or animal pathogenic *S. aureus* (φTGR1), and the PVL-carrying phage strains.

The *S. aureus* Tager 104 genome also indicates that this strain pre-dates the acquisition of the SCC*mec* cassette and may serve to bridge certain clinical MRSA and MSSA strains (Fig. 5 and Additional file 2: Figure S1). Supportive to this hypothesis is the recent observation of an ST49-t208 strain that has become methicillin-resistant [47], potentially through the acquisition of the *mecALGA251* cassette [48]. These results indicate that Tager 104 represents an intermediate departure in the evolution of multi-drug resistant *S. aureus*. Antibiotic susceptibility of *S. aureus* Tager 104 presented here using clinically approved E-test analysis corroborates genomic data (Fig. 8) and indicates that Tager 104 displays a borderline vancomycin intermediate *S. aureus* phenotype (Fig. 8b & e). In fact, current vancomycin guidelines would prohibit use of vancomycin in patients where the isolated strain MIC for vancomycin is equal to or over 2 μg/mL making any potential enhancement to vancomycin resistance or small colony variant formation by Tager 104 could be of significant importance to the understanding of acquired *S. aureus* resistance.

Interestingly, Tager 104 also shows an intermediate adaptation to the nosocomial environment. Tager 104 proteome analysis (Fig. 4) showed an equivalent homology to both community acquired strain SA268 (84.6 %) and nosocomial strain 04–02981 (84.3 %). Therefore, we investigated further the νSaβ genomic island, which has been previously implicated in the shift to hospital settings. Our analysis (Fig. 7) revealed that Tager 104 has lost the genes necessary for competition for resources in the environment, but had yet to gain those necessary for spread in a clinical setting at its time of isolation.

It has been recently suggested that the acquisition of these MGEs may be driven by “glycocodes” encoded by the teichoic acid structure of *S. aureus*, as these structures are recognized by bacteriophages during transduction events [49]. With this in mind, we investigated the locus of teichoic acid biosynthesis in *S. aureus* Tager 104, COL (as a representative of early MRSA strains) and JH1 (as a representative of early VRSA strains) with the hypothesis that homology in these strains indicate a potential phage-mediated acquisition of MGEs. The results indicate that the genomic makeup of this biosynthesis cluster is identical in these three strains (Additional file 5: Figure S3). Therefore, a strain ancestral to Tager 104 acquired resistance elements from bacteriophages, leading to the evolution of more recent clinical MRSA and VRSA strains, in agreement with this phylogenetic analysis (Fig. 5 and Additional file 2: Figure S1). As expected, the PS187 genome described previously showed a very distinct profile, furthering the previous conclusion that this strain is distantly related to other known *S. aureus* lineages. Further analysis will be required to investigate the origin of resistance transfer into Tager 104 and its contribution to *S. aureus* virulence.

## Conclusions

Identification of a suitable reference strain for the study of *S. aureus* pathogenicity is a crucial issue that can give both context and scope to certain single nucleotide polymorphisms present in modern *S. aureus* isolates. Furthermore, the study of the transfer of MGEs between closely related strains may ultimately provide insight into the underlying adaptive pressures that allow these strains to cause disease in a given host. In this article, we have demonstrated that Tager 104 displays a virulent phenotype. Phylogenetic analysis, as well as a set of MGE-encoded factors limited to that which is necessary for systemic survival, suggest that Tager 104 can be used as a reference strain for future studies of *S. aureus* systemic virulence. We have also placed the Tager 104 genome in context with all 75 completed *S. aureus* genomes and shown similarities between Tager 104 genomic islands and other human *S. aureus* pathogens. Moreover, we have established a methodology for the closing of complex genomic repeat regions that may accelerate closure of other genomes in the future, thereby providing an impact to researchers outside the *S. aureus *field. Given the early original isolation of the *S. aureus *Tager 104, the complete characterization presented here should provide new insights into the evolution of this dangerous pathogen.

### Availability of supporting data

The complete Tager 104 genome is available on GenBank as accession number CP012409. Previous scaffolds mentioned in the text may be found on GenBank as accession number AVBR00000001. Phylogenetic data is available at the following URL: http://purl.org/phylo/treebase/phylows/study/TB2:S18752.

## Additional files

Additional file 1: Table S1. Bacterial Strains Used In This Study. (DOCX 212 kb) Additional file 2: Figure S1. Complete Phylogenetic Tree of *S. aureus* Reference Strains. Whole-genome phylogenetic analysis of all *S. aureus* reference strains from Fig. 5. Related branches, such as BAA1680 and the eight ST228 strains had lower bootstrap values, as would be expected given their derivations and regional isolation, respectively. Bootstrap values represent the result of 100 trials. (PDF 180 kb) Additional file 3: Table S2. Genomic Islands and Prophages in the Tager 104 Genome. (DOCX 141 kb) Additional file 4: Figure S2. Homology of Staphylococcal Phage Proteomes to the Tager 104 Prophages. Complete genome sequences were obtained from GenBank for Staphylococcal phages and protein content was predicted using saco_convert on CMG-Biotools 4.3.24. The lists of prophage-encoded proteins for φTGR1 and φTGR2 were obtained from IslandViewer 3. These lists were submitted to BLAST matrix construction on the CMG-Biotools system. (TIF 3029 kb) Additional file 5: Figure S3. Homologous Teichoic Acid Biosynthesis Genes in *S. aureus* Indicate Potential Bacteriophage Interactions. Teichoic acid structure may serve as the mechanism by which phages can recognize and transfer genomic elements. Therefore, investigation of the Tager 104 teichoic acid gene cluster revealed homology to the COL and JH1 strains, confirming the potential of phage-mediated acquisition of genomic elements to radiate into modern clinical strains. Putative proteins are indicated with an asterisk (*). The teichoic acid biosynthesis gene for PS187, which shows homology to transferase genes, is indicated with a double asterisk (**). (TIF 8450 kb)

## Abbreviations

BHI: brain-heart infusion
BLAST: Basic Local Alignment Search Tool
BRIG: BLAST Ring Image Generator
CA-MRSA: community acquired MRSA
CIPRES: Cyberinfrastructure for Phylogenetic Research
ER: erythromycin
H&E: hematoxylin and eosin
HA-MRSA: hospital acquired MRSA
HGAP: Hierarchical Genome Assembly Process
LCB: locally collinear blocks
LZ: linezolid
MGE: mobile genetic elements
MIC: minimum inhibitory concentration
MLST: multi-locus sequence typing
MRSA: methicillin-resistant *S. aureus*
MSSA: methicillin-sensitive *S. aureus*
O.D.: optical density
OCT: optimal cutting temperature
OX: oxacillin
PBS: phosphate buffered saline
PEG: protein-encoding gene
PVL: Panton-Valentine Leukocidin
RAST: Rapid Annotated using Subsystem Technology
*S. aureus*: *Staphylococcus aureus*
SCCmec: Staphylococcal Cassette Chromosome *mec*
SPAdes: St. Petersburg genome assembler
SSPACE: SSAKE-based scaffolding of Pre-Assembled Contigs after Extension
ST: sequence type
TSA: trypticase soy agar
VA: vancomycin
VRSA: vancomycin-resistant *S. aureus*
