# Supplementary material for: Complete genome of Staphylococcus aureus Tager 104 provides evidence of its relation to modern systemic hospital-acquired strains
Source: BMC Genomics. 2016 Mar 3;17:179. doi: 10.1186/s12864-016-2433-8 (PMC4778325; doi:10.1186/s12864-016-2433-8)
Supplement: Additional file 1: Table S1. — Bacterial Strains Used In This Study. (DOCX 212 kb) [file 12864_2016_2433_MOESM1_ESM.docx]

Table S1: Bacterial Strains Used In This Study

| Strain | Date | Location | Host | Infection | ST | References | |
| --- | --- | --- | --- | --- | --- | --- | --- |
| 04-02981 | 2004 | Germany | Human | Not provided | 225 | [1] | |
| 08BA02176 | 2008 | Canada | Human | Wound | 398 | [2] | |
| 11819-97 | 1997 | Denmark | Human | Skin | 80 | [3] | |
| 2395_USA500 | 1994 | USA | Human | Wound | 8 | [4, 5] | |
| 502A | 1963 | USA | Human | Nasal | 5 | [6] | |
| 55/2053 | N/A | N/A | N/A | N/A | 30 | Unpublished | |
| 6850 | 1987 | USA | Human | Skin abscess | 50 | [7] | |
| 71193 | 2004 | USA | Human | Colonization |  | [8, 9] | |
| ATCC 25923 | 1945 | USA | Human | Not provided | 243 | [10] | |
| BAA1680_25b_MRSA | 2003 | USA | Human | Skin | 8 | [11] | |
| BAA1680_26b_MRSA | 2003 | USA | Human | Skin | 8 | [11] | |
| BAA1680_27b_MRSA | 2015 | Laboratory | N/A | N/A | 8 | [11] | |
| BAA1680_29b_MRSA | 2015 | Laboratory | N/A | N/A | 8 | [11] | |
| BAA1680_31b_MRSA | 2015 | Laboratory | N/A | N/A | 8 | [11] | |
| BAA1680_33b | 2015 | Laboratory | N/A | N/A | 8 | [11] | |
| Bmb9393 | 1993 | Brazil | Human | Bloodstream | 239 | [12] | |
| CA-15 | 2007 | Colombia | Human | Not provided | 8* | [13] | |
| CA-347 | 2005 | USA | Human | Bloodstream | 45 | [14] | |
| CN1 | 2006 | South Korea | Human | Necrotizing fasciitis | 72 | [15] | |
| COL | 1960 | UK | Human | Surgical | 250 | [16] | |
| DSM 20231 | 1884 | Germany | Human | Pleural fluid | 8 | [17] | |
| ECT-R2 | 2004 | Sweden | Human | Not provided | 5 | [18] | |
| ED133 | 1997 | France | Ovine | Mastitis | 133 | [19, 20] | |
| E-MRSA-15 | N/A | UK | Human | Bloodstream | 22 | [21] | |
| FCFHV36 | 2015 | Brazil | Human | Bone | 105 | [22] | |
| FORC_001 | N/A | South Korea | N/A | N/A (soy bean) | 30 | [23] | |
| GR2 | 2006 | Greece | Human | Nasal | 728* | [24] | |
| Gv69 | N/A | N/A | N/A | N/A | 239 | Unpublished | |
| HO 5096 0412 | 2005 | UK | Human | Neonatal | 22 | [25] | |
| HOU1444-VR | 2012 | Brazil | Human | Bloodstream | 5 | [26] | |
| ILRI_Eymole1/1 | N/A | N/A | N/A | N/A | 30 | Unpublished | |
| JH1 | 2001 | United States | Human | Bloodstream | 105 | [27] | |
| JH9 | 2001 | United States | Human | Bloodstream | 105 | [27] | |
| JKD6008 | 2003 | New Zealand | Human | Bloodstream | 239 | [28] | |
| JKD6159 | 2004 | Australia | Human | Sepsis | 93 | [29] | |
| JS395 | N/A | N/A | N/A | N/A | 1093 | Unpublished | |
| M013 | 2002 | Taiwan | Human | Wound | 59 | [30] | |
| M121 | 2006 | Colombia | Not provided | Not provided | 8 | [13] | |
| MRSA252 | 1997 | UK | Human | Sepsis | 36 | [31] | |
| MSHR1132 | 2006 | Australia | Human | Necrotizing fasciitis |  | [32] | |
| MSSA476 | 1998 | UK | Human | Bone | 1 | [31] |  |
| Mu3 | 1996 | Japan | Human | Pneumonia | 5 | [33, 34] |  |
| Mu50 | 1997 | Japan | Human | Surgical | 5 | [35] |  |
| MW2 | 1998 | USA | Human | Septicemia | 1 | [36] |  |
| N315 | 1982 | Japan | Human | Throat | 5 | [35] |  |
| NCTC8325 | 1960 | UK | Human | Conjunctiva | 8 | [37] |  |
| Newman | 1954 | UK | Human | Osteomyelitis | 254 | [38] |  |
| NRS 100 | 1960 | UK | Human | Surgical | 250 | Unpublished |  |
| RF122 | 1993 | Ireland | Bovine | Mastitis | 151 | [39, 40] |  |
| RKI4 | 2008 | Germany | Human | Gastric | 27 | [41] |  |
| S0385 | 2006 | Netherlands | Human | Endocarditis | 395** | [42] |  |
| SA268 | 2012 | China | Human | Sepsis | 59 | [43] |  |
| SA40 | 2005 | Taiwan | Human | Nasal | 59* | [15] |  |
| SA564 | 2013 | Switzerland | Human | Bloodstream | 5 | [44] |  |
| SA957 | 2000 | Taiwan | Human | Bloodstream | 59 | [15] |  |
| ST228_10388 | 2001 | Switzerland | Human | Not provided | 228 | [45] |  |
| ST228_10497 | 2001 | Switzerland | Human | Not provided | 228 | [45] |  |
| ST228_15532 | 2006 | Switzerland | Human | Not provided | 228 | [45] |  |
| ST228_16035 | 2006 | Switzerland | Human | Not provided | 228 | [45] |  |
| ST228_16125 | 2006 | Switzerland | Human | Not provided | 228 | [45] |  |
| ST228_18341 | 2008 | Switzerland | Human | Not provided | 228 | [45] |  |
| ST228_18412 | 2008 | Switzerland | Human | Not provided | 228 | [45] |  |
| ST228_18583 | 2008 | Switzerland | Human | Not provided | 228 | [45] |  |
| DAR4145 | 2009 | India | Human | Abscess | 772 | [46, 47] |  |
| T0131 | 2006 | China | Human | Not provided | 239 | [48] |  |
| Tager 104 | 1947 | USA | Human | Impetigo | 49 | This paper |  |
| TCH60 | 2008 | USA | Human | Skin | 30* | ATCC |  |
| TW20 | 2003 | UK | Human | Bacteremia | 239 | [49] |  |
| UA-S391_USA300 | N/A | USA | N/A | N/A | 8 | [21] |  |
| USA300_2014.C01 | 2014 | USA | Human | Wound | 8 | [50] |  |
| USA300_2014.C02 | 2014 | USA | Human | Wound | 8 | [50] |  |
| USA300_FPR3757 | 2004 | USA | Human | Abscess | 8 | [51, 52] |  |
| USA300_TCH1516 | 2004 | USA | Human | Bacteremia | 8 | [53, 54] |  |
| USA300-ISMMS1 | 2014 | USA | Human | Bacteremia | 8 | [55] |  |
| VC40 | 2002 | Laboratory | N/A | N/A | 8 | [56] |  |
| Z172 | 2010 | Taiwan | Human | Bacteremia | 239 | [57] |  |

**denotes strain genomes was completed by Oct. 2015 but ST was not determined by automated CLC software.*

***denoted strain genomes was not completed by Oct. 2015 and ST was not determined by automated CLC software.*

**SUPPLEMENTAL TABLE REFERENCES**

1. Nubel U, Dordel J, Kurt K, Strommenger B, Westh H, Shukla SK, Zemlickova H, Leblois R, Wirth T, Jombart T *et al*: **A timescale for evolution, population expansion, and spatial spread of an emerging clone of methicillin-resistant Staphylococcus aureus**. *PLoS Pathog* 2010, **6**(4):e1000855.

2. Golding GR, Bryden L, Levett PN, McDonald RR, Wong A, Graham MR, Tyler S, Van Domselaar G, Mabon P, Kent H *et al*: **whole-genome sequence of livestock-associated st398 methicillin-resistant staphylococcus aureus Isolated from Humans in Canada**. *J Bacteriol* 2012, **194**(23):6627-6628.

3. Stegger M, Price LB, Larsen AR, Gillece JD, Waters AE, Skov R, Andersen PS: **Genome sequence of Staphylococcus aureus strain 11819-97, an ST80-IV European community-acquired methicillin-resistant isolate**. *J Bacteriol* 2012, **194**(6):1625-1626.

4. Benson MA, Ohneck EA, Ryan C, Alonzo F, 3rd, Smith H, Narechania A, Kolokotronis SO, Satola SW, Uhlemann AC, Sebra R *et al*: **Evolution of hypervirulence by a MRSA clone through acquisition of a transposable element**. *Mol Microbiol* 2014, **93**(4):664-681.

5. Roberts RB, de Lencastre A, Eisner W, Severina EP, Shopsin B, Kreiswirth BN, Tomasz A: **Molecular epidemiology of methicillin-resistant Staphylococcus aureus in 12 New York hospitals. MRSA Collaborative Study Group**. *J Infect Dis* 1998, **178**(1):164-171.

6. Parker D, Narechania A, Sebra R, Deikus G, Larussa S, Ryan C, Smith H, Prince A, Mathema B, Ratner AJ *et al*: **Genome Sequence of Bacterial Interference Strain Staphylococcus aureus 502A**. *Genome Announc* 2014, **2**(2).

7. Fraunholz M, Bernhardt J, Schuldes J, Daniel R, Hecker M, Sinha B: **Complete Genome Sequence of Staphylococcus aureus 6850, a Highly Cytotoxic and Clinically Virulent Methicillin-Sensitive Strain with Distant Relatedness to Prototype Strains**. *Genome Announc* 2013, **1**(5).

8. Bhat M, Dumortier C, Taylor BS, Miller M, Vasquez G, Yunen J, Brudney K, Sanchez EJ, Rodriguez-Taveras C, Rojas R *et al*: **Staphylococcus aureus ST398, New York City and Dominican Republic**. *Emerg Infect Dis* 2009, **15**(2):285-287.

9. Uhlemann AC, Porcella SF, Trivedi S, Sullivan SB, Hafer C, Kennedy AD, Barbian KD, McCarthy AJ, Street C, Hirschberg DL *et al*: **Identification of a highly transmissible animal-independent Staphylococcus aureus ST398 clone with distinct genomic and cell adhesion properties**. *MBio* 2012, **3**(2).

10. Treangen TJ, Maybank RA, Enke S, Friss MB, Diviak LF, Karaolis DK, Koren S, Ondov B, Phillippy AM, Bergman NH *et al*: **Complete Genome Sequence of the Quality Control Strain Staphylococcus aureus subsp. aureus ATCC 25923**. *Genome Announc* 2014, **2**(6).

11. Daum LT, Bumah VV, Masson-Meyers DS, Khubbar M, Rodriguez JD, Fischer GW, Enwemeka CS, Gradus S, Bhattacharyya S: **Whole-Genome Sequence for Methicillin-Resistant Staphylococcus aureus Strain ATCC BAA-1680**. *Genome Announc* 2015, **3**(2).

12. Qu JQ, Liu C, Wang XM, Zhang ZB, Chi S, Liu T: **Complete mitochondrial genome of Costaria costata shows conservative evolution in Laminariales**. *Mitochondrial DNA* 2015, **26**(6):919-920.

13. Planet PJ, Diaz L, Kolokotronis SO, Narechania A, Reyes J, Xing G, Rincon S, Smith H, Panesso D, Ryan C *et al*: **Parallel Epidemics of Community-Associated Methicillin-Resistant Staphylococcus aureus USA300 Infection in North and South America**. *J Infect Dis* 2015, **212**(12):1874-1882.

14. Stegger M, Driebe EM, Roe C, Lemmer D, Bowers JR, Engelthaler DM, Keim P, Andersen PS: **Genome Sequence of Staphylococcus aureus Strain CA-347, a USA600 Methicillin-Resistant Isolate**. *Genome Announc* 2013, **1**(4).

15. Chen CJ, Unger C, Hoffmann W, Lindsay JA, Huang YC, Gotz F: **Characterization and comparison of 2 distinct epidemic community-associated methicillin-resistant Staphylococcus aureus clones of ST59 lineage**. *PLoS One* 2013, **8**(9):e63210.

16. Gill SR, Fouts DE, Archer GL, Mongodin EF, Deboy RT, Ravel J, Paulsen IT, Kolonay JF, Brinkac L, Beanan M *et al*: **Insights on evolution of virulence and resistance from the complete genome analysis of an early methicillin-resistant Staphylococcus aureus strain and a biofilm-producing methicillin-resistant Staphylococcus epidermidis strain**. *J Bacteriol* 2005, **187**(7):2426-2438.

17. Shiroma A, Terabayashi Y, Nakano K, Shimoji M, Tamotsu H, Ashimine N, Ohki S, Shinzato M, Teruya K, Satou K *et al*: **First Complete Genome Sequences of Staphylococcus aureus subsp. aureus Rosenbach 1884 (DSM 20231T), Determined by PacBio Single-Molecule Real-Time Technology**. *Genome Announc* 2015, **3**(4).

18. Lindqvist M, Isaksson B, Grub C, Jonassen TO, Hallgren A: **Detection and characterisation of SCCmec remnants in multiresistant methicillin-susceptible Staphylococcus aureus causing a clonal outbreak in a Swedish county**. *Eur J Clin Microbiol Infect Dis* 2012, **31**(2):141-147.

19. Ben Zakour NL, Sturdevant DE, Even S, Guinane CM, Barbey C, Alves PD, Cochet MF, Gautier M, Otto M, Fitzgerald JR *et al*: **Genome-wide analysis of ruminant Staphylococcus aureus reveals diversification of the core genome**. *J Bacteriol* 2008, **190**(19):6302-6317.

20. Guinane CM, Ben Zakour NL, Tormo-Mas MA, Weinert LA, Lowder BV, Cartwright RA, Smyth DS, Smyth CJ, Lindsay JA, Gould KA *et al*: **Evolutionary genomics of Staphylococcus aureus reveals insights into the origin and molecular basis of ruminant host adaptation**. *Genome Biol Evol* 2010, **2**:454-466.

21. Sabirova JS, Xavier BB, Hernalsteens JP, De Greve H, Ieven M, Goossens H, Malhotra-Kumar S: **Complete Genome Sequences of Two Prolific Biofilm-Forming Staphylococcus aureus Isolates Belonging to USA300 and EMRSA-15 Clonal Lineages**. *Genome Announc* 2014, **2**(3).

22. McCulloch JA, Silveira AC, Lima Moraes Ada C, Perez-Chaparro PJ, Ferreira Silva M, Almeida LM, d'Azevedo PA, Mamizuka EM: **Complete Genome Sequence of Staphylococcus aureus FCFHV36, a Methicillin-Resistant Strain Heterogeneously Resistant to Vancomycin**. *Genome Announc* 2015, **3**(4).

23. Lim S, Lee DH, Kwak W, Shin H, Ku HJ, Lee JE, Lee GE, Kim H, Choi SH, Ryu S *et al*: **Comparative genomic analysis of Staphylococcus aureus FORC_001 and S. aureus MRSA252 reveals the characteristics of antibiotic resistance and virulence factors for human infection**. *J Microbiol Biotechnol* 2015, **25**(1):98-108.

24. Sabat AJ, Pournaras S, Akkerboom V, Tsakris A, Grundmann H, Friedrich AW: **Whole-genome analysis of an oxacillin-susceptible CC80 mecA-positive Staphylococcus aureus clinical isolate: insights into the mechanisms of cryptic methicillin resistance**. *J Antimicrob Chemother* 2015, **70**(11):2956-2964.

25. Holden MT, Hsu LY, Kurt K, Weinert LA, Mather AE, Harris SR, Strommenger B, Layer F, Witte W, de Lencastre H *et al*: **A genomic portrait of the emergence, evolution, and global spread of a methicillin-resistant Staphylococcus aureus pandemic**. *Genome Res* 2013, **23**(4):653-664.

26. Panesso D, Planet PJ, Diaz L, Hugonnet JE, Tran TT, Narechania A, Munita JM, Rincon S, Carvajal LP, Reyes J *et al*: **Methicillin-Susceptible, Vancomycin-Resistant Staphylococcus aureus, Brazil**. *Emerg Infect Dis* 2015, **21**(10):1844-1848.

27. Sieradzki K, Leski T, Dick J, Borio L, Tomasz A: **Evolution of a vancomycin-intermediate Staphylococcus aureus strain in vivo: multiple changes in the antibiotic resistance phenotypes of a single lineage of methicillin-resistant S. aureus under the impact of antibiotics administered for chemotherapy**. *J Clin Microbiol* 2003, **41**(4):1687-1693.

28. Howden BP, Seemann T, Harrison PF, McEvoy CR, Stanton JA, Rand CJ, Mason CW, Jensen SO, Firth N, Davies JK *et al*: **Complete genome sequence of Staphylococcus aureus strain JKD6008, an ST239 clone of methicillin-resistant Staphylococcus aureus with intermediate-level vancomycin resistance**. *J Bacteriol* 2010, **192**(21):5848-5849.

29. Chua K, Seemann T, Harrison PF, Davies JK, Coutts SJ, Chen H, Haring V, Moore R, Howden BP, Stinear TP: **Complete genome sequence of Staphylococcus aureus strain JKD6159, a unique Australian clone of ST93-IV community methicillin-resistant Staphylococcus aureus**. *J Bacteriol* 2010, **192**(20):5556-5557.

30. Huang TW, Chen FJ, Miu WC, Liao TL, Lin AC, Huang IW, Wu KM, Tsai SF, Chen YT, Lauderdale TL: **Complete genome sequence of Staphylococcus aureus M013, a pvl-positive, ST59-SCCmec type V strain isolated in Taiwan**. *J Bacteriol* 2012, **194**(5):1256-1257.

31. Holden MT, Feil EJ, Lindsay JA, Peacock SJ, Day NP, Enright MC, Foster TJ, Moore CE, Hurst L, Atkin R *et al*: **Complete genomes of two clinical Staphylococcus aureus strains: evidence for the rapid evolution of virulence and drug resistance**. *Proc Natl Acad Sci U S A* 2004, **101**(26):9786-9791.

32. Holt DC, Holden MT, Tong SY, Castillo-Ramirez S, Clarke L, Quail MA, Currie BJ, Parkhill J, Bentley SD, Feil EJ *et al*: **A very early-branching Staphylococcus aureus lineage lacking the carotenoid pigment staphyloxanthin**. *Genome Biol Evol* 2011, **3**:881-895.

33. Hiramatsu K, Aritaka N, Hanaki H, Kawasaki S, Hosoda Y, Hori S, Fukuchi Y, Kobayashi I: **Dissemination in Japanese hospitals of strains of Staphylococcus aureus heterogeneously resistant to vancomycin**. *Lancet* 1997, **350**(9092):1670-1673.

34. Liu C, Chambers HF: **Staphylococcus aureus with heterogeneous resistance to vancomycin: epidemiology, clinical significance, and critical assessment of diagnostic methods**. *Antimicrob Agents Chemother* 2003, **47**(10):3040-3045.

35. Kuroda M, Ohta T, Uchiyama I, Baba T, Yuzawa H, Kobayashi I, Cui L, Oguchi A, Aoki K, Nagai Y *et al*: **Whole genome sequencing of meticillin-resistant Staphylococcus aureus**. *Lancet* 2001, **357**(9264):1225-1240.

36. Baba T, Takeuchi F, Kuroda M, Yuzawa H, Aoki K, Oguchi A, Nagai Y, Iwama N, Asano K, Naimi T *et al*: **Genome and virulence determinants of high virulence community-acquired MRSA**. *Lancet* 2002, **359**(9320):1819-1827.

37. Herbert S, Ziebandt AK, Ohlsen K, Schafer T, Hecker M, Albrecht D, Novick R, Gotz F: **Repair of global regulators in Staphylococcus aureus 8325 and comparative analysis with other clinical isolates**. *Infect Immun* 2010, **78**(6):2877-2889.

38. Baba T, Bae T, Schneewind O, Takeuchi F, Hiramatsu K: **Genome sequence of Staphylococcus aureus strain Newman and comparative analysis of staphylococcal genomes: polymorphism and evolution of two major pathogenicity islands**. *J Bacteriol* 2008, **190**(1):300-310.

39. Herron-Olson L, Fitzgerald JR, Musser JM, Kapur V: **Molecular correlates of host specialization in Staphylococcus aureus**. *PLoS One* 2007, **2**(10):e1120.

40. Fitzgerald JR, Meaney WJ, Hartigan PJ, Smyth CJ, Kapur V: **Fine-structure molecular epidemiological analysis of Staphylococcus aureus recovered from cows**. *Epidemiol Infect* 1997, **119**(2):261-269.

41. Stevens MJ, Stephan R, Johler S: **Complete and Assembled Genome Sequence of Staphylococcus aureus RKI4, a Food-Poisoning Strain Exhibiting a Novel S. aureus Pathogenicity Island Carrying seb**. *Genome Announc* 2015, **3**(4).

42. Schijffelen MJ, Boel CH, van Strijp JA, Fluit AC: **Whole genome analysis of a livestock-associated methicillin-resistant Staphylococcus aureus ST398 isolate from a case of human endocarditis**. *BMC Genomics* 2010, **11**:376.

43. Qu T, Feng Y, Jiang Y, Zhu P, Wei Z, Chen Y, Otto M, Yu Y: **Whole genome analysis of a community-associated methicillin-resistant Staphylococcus aureus ST59 isolate from a case of human sepsis and severe pneumonia in China**. *PLoS One* 2014, **9**(2):e89235.

44. Giraud C, Hausmann S, Lemeille S, Prados J, Redder P, Linder P: **The C-terminal region of the RNA helicase CshA is required for the interaction with the degradosome and turnover of bulk RNA in the opportunistic pathogen Staphylococcus aureus**. *RNA Biol* 2015, **12**(6):658-674.

45. Vogel V, Falquet L, Calderon-Copete SP, Basset P, Blanc DS: **Short term evolution of a highly transmissible methicillin-resistant Staphylococcus aureus clone (ST228) in a tertiary care hospital**. *PLoS One* 2012, **7**(6):e38969.

46. Steinig EJ, Andersson P, Harris SR, Sarovich DS, Manoharan A, Coupland P, Holden MT, Parkhill J, Bentley SD, Robinson DA *et al*: **Single-molecule sequencing reveals the molecular basis of multidrug-resistance in ST772 methicillin-resistant Staphylococcus aureus**. *BMC Genomics* 2015, **16**:388.

47. Manoharan A, Zhang L, Poojary A, Bhandarkar L, Koppikar G, Robinson DA: **An outbreak of post-partum breast abscesses in Mumbai, India caused by ST22-MRSA-IV: genetic characteristics and epidemiological implications**. *Epidemiol Infect* 2012, **140**(10):1809-1812.

48. Li Y, Cao B, Zhang Y, Zhou J, Yang B, Wang L: **Complete genome sequence of Staphylococcus aureus T0131, an ST239-MRSA-SCCmec type III clone isolated in China**. *J Bacteriol* 2011, **193**(13):3411-3412.

49. Harris SR, Feil EJ, Holden MT, Quail MA, Nickerson EK, Chantratita N, Gardete S, Tavares A, Day N, Lindsay JA *et al*: **Evolution of MRSA during hospital transmission and intercontinental spread**. *Science* 2010, **327**(5964):469-474.

50. Johnson RC, Schlett CD, Crawford K, Lanier JB, Merrell DS, Ellis MW: **Recurrent Methicillin-Resistant Staphylococcus aureus Cutaneous Abscesses and Selection of Reduced Chlorhexidine Susceptibility during Chlorhexidine Use**. *J Clin Microbiol* 2015, **53**(11):3677-3682.

51. Diep BA, Chambers HF, Graber CJ, Szumowski JD, Miller LG, Han LL, Chen JH, Lin F, Lin J, Phan TH *et al*: **Emergence of multidrug-resistant, community-associated, methicillin-resistant Staphylococcus aureus clone USA300 in men who have sex with men**. *Ann Intern Med* 2008, **148**(4):249-257.

52. Diep BA, Gill SR, Chang RF, Phan TH, Chen JH, Davidson MG, Lin F, Lin J, Carleton HA, Mongodin EF *et al*: **Complete genome sequence of USA300, an epidemic clone of community-acquired meticillin-resistant Staphylococcus aureus**. *Lancet* 2006, **367**(9512):731-739.

53. Highlander SK, Hulten KG, Qin X, Jiang H, Yerrapragada S, Mason EO, Jr., Shang Y, Williams TM, Fortunov RM, Liu Y *et al*: **Subtle genetic changes enhance virulence of methicillin resistant and sensitive Staphylococcus aureus**. *BMC Microbiol* 2007, **7**:99.

54. Gonzalez BE, Martinez-Aguilar G, Hulten KG, Hammerman WA, Coss-Bu J, Avalos-Mishaan A, Mason EO, Jr., Kaplan SL: **Severe Staphylococcal sepsis in adolescents in the era of community-acquired methicillin-resistant Staphylococcus aureus**. *Pediatrics* 2005, **115**(3):642-648.

55. Altman DR, Sebra R, Hand J, Attie O, Deikus G, Carpini KW, Patel G, Rana M, Arvelakis A, Grewal P *et al*: **Transmission of methicillin-resistant Staphylococcus aureus via deceased donor liver transplantation confirmed by whole genome sequencing**. *Am J Transplant* 2014, **14**(11):2640-2644.

56. Schaaff F, Reipert A, Bierbaum G: **An elevated mutation frequency favors development of vancomycin resistance in Staphylococcus aureus**. *Antimicrob Agents Chemother* 2002, **46**(11):3540-3548.

57. Chen FJ, Lauderdale TL, Wang LS, Huang IW: **Complete Genome Sequence of Staphylococcus aureus Z172, a Vancomycin-Intermediate and Daptomycin-Nonsusceptible Methicillin-Resistant Strain Isolated in Taiwan**. *Genome Announc* 2013, **1**(6).
