# Supplementary figures and images for: Complete genome of Staphylococcus aureus Tager 104 provides evidence of its relation to modern systemic hospital-acquired strains
Source: BMC Genomics. 2016 Mar 3;17:179. doi: 10.1186/s12864-016-2433-8 (PMC4778325; doi:10.1186/s12864-016-2433-8)

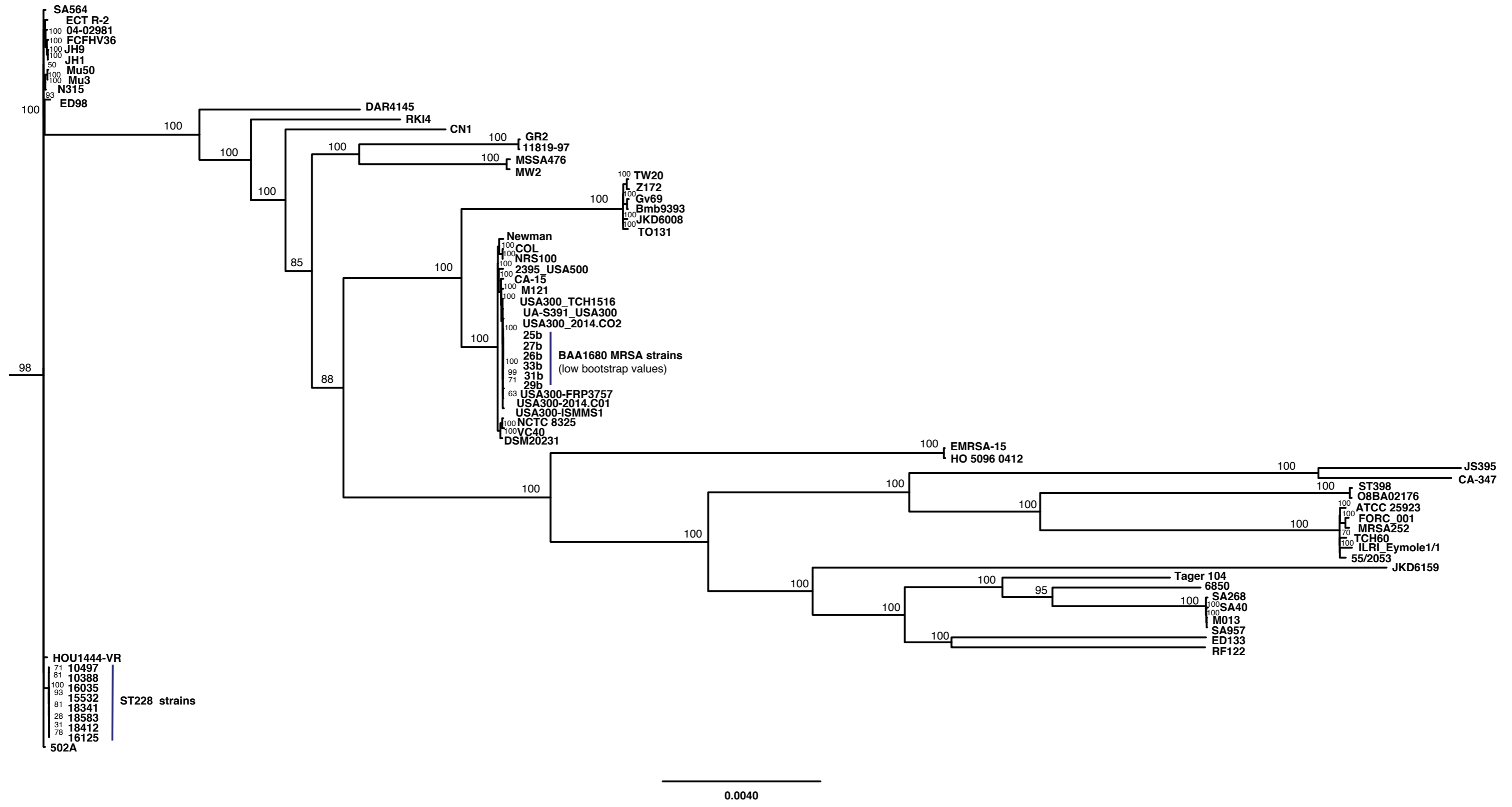

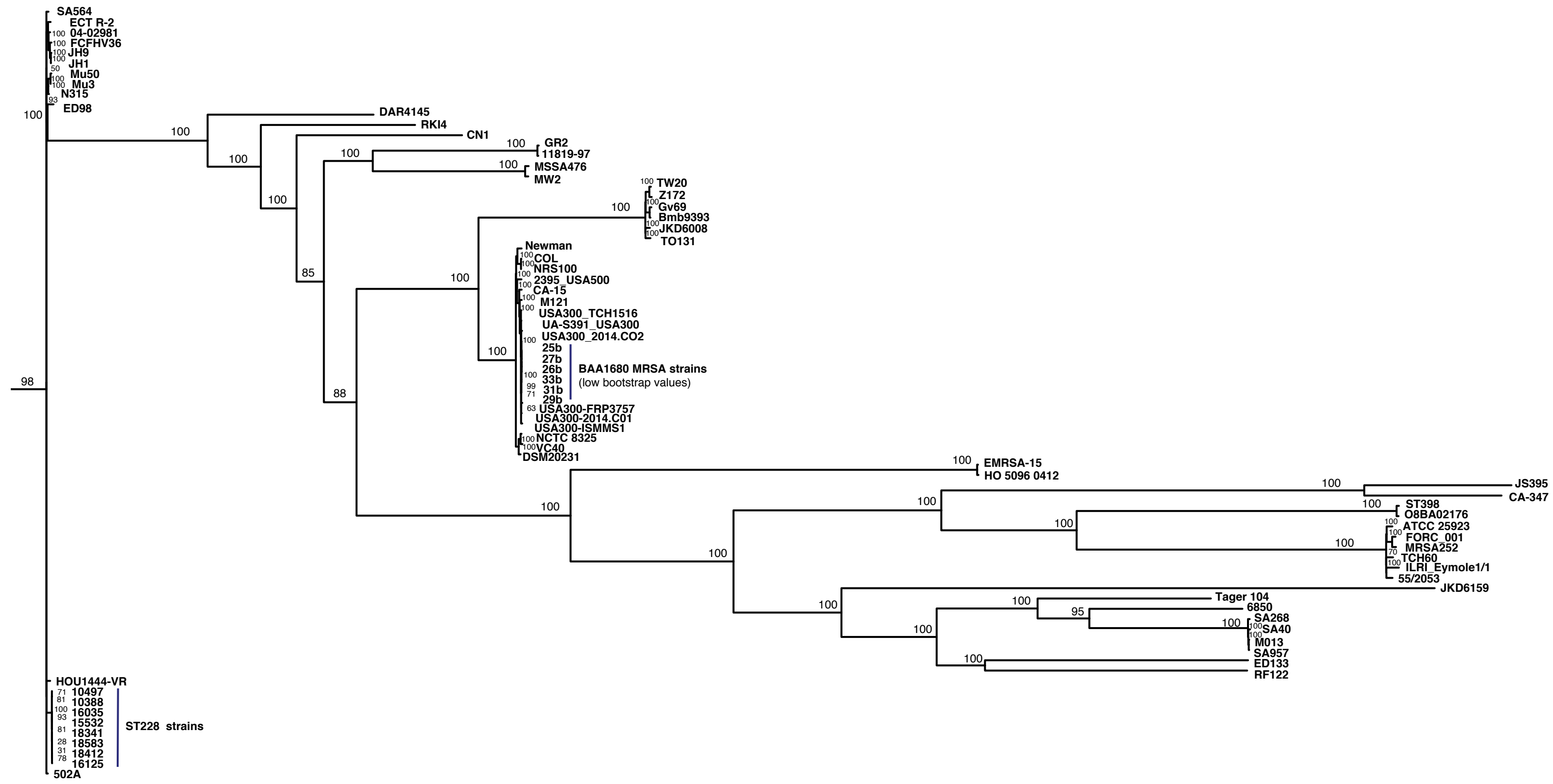

0.0040

Supplement: Additional file 2: Figure S1. — Complete Phylogenetic Tree of S. aureus Reference Strains. Whole-genome phylogenetic analysis of all S. aureus reference strains from Fig. 5. Related branches, such as BAA1680 and the eight ST228 strains had lower bootstrap values, as would be expected given their derivations and regional isolation, respectively. Bootstrap values represent the result of 100 trials. (PDF 180 kb) [file 12864_2016_2433_MOESM2_ESM.pdf]

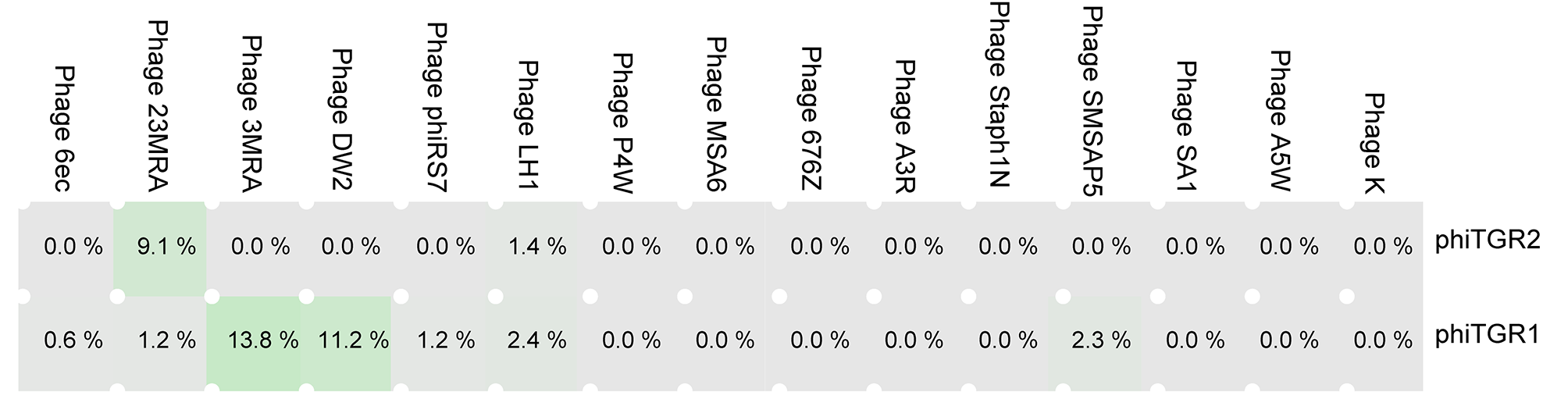

Supplement: Additional file 4: Figure S2. — Homology of Staphylococcal Phage Proteomes to the Tager 104 Prophages. Complete genome sequences were obtained from GenBank for Staphylococcal phages and protein content was predicted using saco_convert on CMG-Biotools 4.3.24. The lists of prophage-encoded proteins for φTGR1 and φTGR2 were obtained from IslandViewer 3. These lists were submitted to BLAST matrix construction on the CMG-Biotools system. (TIF 3029 kb) [file 12864_2016_2433_MOESM4_ESM.tif]

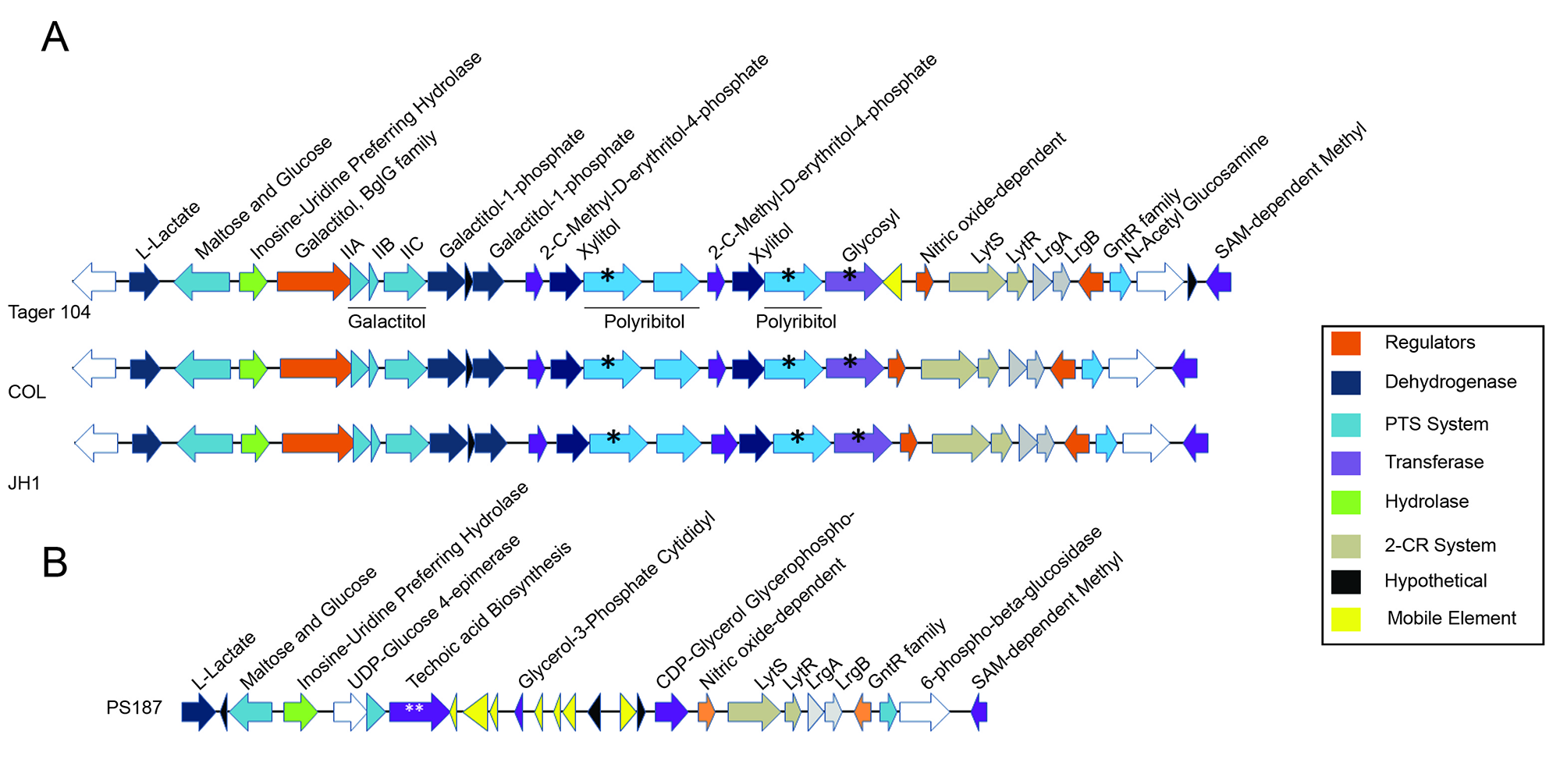

Supplement: Additional file 5: Figure S3. — Homologous Teichoic Acid Biosynthesis Genes in S. aureus Indicate Potential Bacteriophage Interactions. Teichoic acid structure may serve as the mechanism by which phages can recognize and transfer genomic elements. Therefore, investigation of the Tager 104 teichoic acid gene cluster revealed homology to the COL and JH1 strains, confirming the potential of phage-mediated acquisition of genomic elements to radiate into modern clinical strains. Putative proteins are indicated with an asterisk (*). The teichoic acid biosynthesis gene for PS187, which shows homology to transferase genes, is indicated with a double asterisk (**). (TIF 8450 kb) [file 12864_2016_2433_MOESM5_ESM.tif]
