# Supplementary material for: Complete genome of Staphylococcus aureus Tager 104 provides evidence of its relation to modern systemic hospital-acquired strains
Source: BMC Genomics. 2016 Mar 3;17:179. doi: 10.1186/s12864-016-2433-8 (PMC4778325; doi:10.1186/s12864-016-2433-8)
Supplement: Additional file 3: Table S2. — Genomic Islands and Prophages in the Tager 104 Genome. (DOCX 141 kb) [file 12864_2016_2433_MOESM3_ESM.docx]

Table S2: Genomic Islands and Prophages in the Tager 104 Genome

| Genomic Location | Name | BLAST  Result | Gene Annotations |
| --- | --- | --- | --- |
| GENOMIC ISLANDS | | | |
| 1,165,336 - 1,170,248 | TGR1 | SA268, SA40, SA957, M013 | ORF031 |
|  |  |  | ORF021 |
|  |  |  | Phage lysin, N-acetylmuramoyl-L-alanine amidase (EC 3.5.1.28) |
|  |  |  | Phage holin |
|  |  |  | Phage protein |
|  |  |  | Tail fiber protein [SA bacteriophages 11, Mu50B] |
|  |  |  | Phage lysin, N-acetylmuramoyl-L-alanine amidase (EC 3.5.1.28) |
|  |  |  | Phage protein |
|  |  |  | Hypothetical protein, phi-ETA orf58 homolog [SA bacteriophages 11, Mu50B] |
|  |  |  | Phage protein |
|  |  |  | FIG01108548: hypothetical protein |
|  |  |  | Putative major teichoic acid biosynthesis protein C |
|  |  |  | Phage minor structural protein |
|  |  |  | phi 11 orf43 homolog [SA bacteriophages 11, Mu50B] |
|  |  |  | Phage tail length tape-measure protein |
|  |  |  | phi 11 orf41 homolog [SA bacteriophages 11, Mu50B] |
|  |  |  | Phage protein |
|  |  |  | Phage tail protein |
|  |  |  | Phage protein |
|  |  |  | Phage protein |
|  |  |  | Phage protein |
|  |  |  | Phage transcriptional terminator |
|  |  |  | Phage major capsid protein |
|  |  |  | Phage capsid and scaffold |
| 1,192,427 - 1,201,074 | TGR2 | 6850 | FIG01108790: hypothetical protein |
|  |  |  | FIG01108566: hypothetical protein |
|  |  |  | FIG01107981: hypothetical protein |
|  |  |  | Integrase, superantigen-encoding pathogenicity islands SaPI |
|  |  |  | FIG01108853: hypothetical protein |
|  |  |  | FIG01108840: hypothetical protein |
|  |  |  | hypothetical protein |
|  |  |  | hypothetical protein |
|  |  |  | hypothetical protein |
|  |  |  | hypothetical protein |
|  |  |  | hypothetical protein |
|  |  |  | hypothetical protein |
|  |  |  | Putative terminase, superantigen-encoding pathogenicity islands SaPI |
|  |  |  | Hypothetical SAV0799 homolog in superantigen-encoding pathogenicity islands SaPI |
| 1,285,693 - 1,291,359 | TGR3 | SA40, SA957, M013 | FIG01108751: hypothetical protein |
|  |  |  | FIG01108312: hypothetical protein |
|  |  |  | FIG01108876: hypothetical protein |
|  |  |  | FIG01107881: hypothetical protein |
|  |  |  | Lmo0069 homolog within ESAT-6 gene cluster |
|  |  |  | Lmo0069 homolog within ESAT-6 gene cluster |
|  |  |  | FIG01108656: hypothetical protein |
|  |  |  | FIG01108452: hypothetical protein |
|  |  |  | FtsK/SpoIIIE family protein, putative EssC component of Type VII secretion system |
| 1,523,588 - 1,529,604 | TGR4 | SA957 | acetyltransferase (GNAT) family protein |
|  |  |  | HTH-type transcriptional regulator LrpC |
|  |  |  | hypothetical protein |
|  |  |  | hypothetical protein |
|  |  |  | Bipolar DNA helicase HerA |
|  |  |  | FIG036446: hypothetical protein |
|  |  |  | hypothetical protein |
| 2,170,899 - 2,174,905 | TGR5 | FORC_001, MRSA252 | FIG01107943: hypothetical protein |
|  |  |  | FIG01107943: hypothetical protein |
|  |  |  | FIG01107943: hypothetical protein |
|  |  |  | Transcriptional regulator, Cro/CI family protein transposon-related |
|  |  |  | FIG01107943: hypothetical protein |
|  |  |  | FIG01107943: hypothetical protein |
|  |  |  | FIG01107943: hypothetical protein |

|  |  |  |  |
| --- | --- | --- | --- |
| Prophages | | | |
| 447,213 - 490,078 | ϕTGR1 | ϕNM1-4 | ORF031 |
|  |  |  | ORF021 |
|  |  |  | Phage lysin, N-acetylmuramoyl-L-alanine amidase (EC 3.5.1.28) |
|  |  |  | Phage holin |
|  |  |  | Phage protein |
|  |  |  | Tail fiber protein [SA bacteriophages 11, Mu50B] |
|  |  |  | Phage lysin, N-acetylmuramoyl-L-alanine amidase (EC 3.5.1.28) |
|  |  |  | Phage protein |
|  |  |  | Hypothetical protein, phi-ETA orf58 homolog [SA bacteriophages 11, Mu50B] |
|  |  |  | Phage protein |
|  |  |  | FIG01108548: hypothetical protein |
|  |  |  | Putative major teichoic acid biosynthesis protein C |
|  |  |  | Phage minor structural protein |
|  |  |  | phi 11 orf43 homolog [SA bacteriophages 11, Mu50B] |
|  |  |  | Phage tail length tape-measure protein |
|  |  |  | phi 11 orf41 homolog [SA bacteriophages 11, Mu50B] |
|  |  |  | Phage protein |
|  |  |  | Phage tail protein |
|  |  |  | Phage protein |
|  |  |  | Phage protein |
|  |  |  | Phage protein |
|  |  |  | Phage transcriptional terminator |
|  |  |  | Phage major capsid protein |
|  |  |  | Phage capsid and scaffold |
|  |  |  | Phage protein |
|  |  |  | Phage protein |
|  |  |  | Phage portal protein |
|  |  |  | Phage terminase, large subunit |
|  |  |  | Phage terminase, small subunit |
|  |  |  | Integrase regulator RinA |
|  |  |  | Phage protein |
|  |  |  | Transcriptional activator rinB, phage associated |
|  |  |  | Hypothetical protein, SAV0877 homolog [SA bacteriophages 11, Mu50B] |
|  |  |  | hypothetical protein |
|  |  |  | ORF058 |
|  |  |  | Phage protein |
|  |  |  | ORF077 |
|  |  |  | Phage antirepressor protein |
|  |  |  | Cro-like repressor [SA bacteriophages 11, Mu50B] |
|  |  |  | Phage repressor |
|  |  |  | hypothetical protein within prophage |
|  |  |  | Hypothetical protein, SAV0849 homolog [SA bacteriophages 11, Mu50B] |
|  |  |  | Phage excisionase |
|  |  |  | Phage DNA invertase |
| 2,308,294 - 2,313,700 | ϕPVL or other | | Acetylornithine deacetylase (EC 3.5.1.16) |
|  |  |  | Leukocidin LukS-PV |
|  |  |  | Leukocidin LukF-PV |
|  |  |  | Beta-hemolysin |
|  |  |  | Phage integrase |
| 2,313,771 - 2,320,128 | ϕTGR2 | ϕNM3 | Phage integrase |
|  |  |  | Phage protein |
|  |  |  | glycosyl transferase |
|  |  |  | hypothetical protein within prophage |
|  |  |  | Phage protein |
|  |  |  | DNA helicase, phage-associated |
|  |  |  | Phage repressor |
|  |  |  | DNA-binding protein, phage associated |
|  |  |  | hypothetical protein within prophage |
|  |  |  | hypothetical protein within prophage |
|  |  |  | Phage antirepressor protein |
